# P2X7 Receptor Promotes Mouse Mammary Cancer Cell Invasiveness and Tumour Progression, and Is a Target for Anticancer Treatment

**DOI:** 10.3390/cancers12092342

**Published:** 2020-08-19

**Authors:** Lucie Brisson, Stéphanie Chadet, Osbaldo Lopez-Charcas, Bilel Jelassi, David Ternant, Julie Chamouton, Stéphanie Lerondel, Alain Le Pape, Isabelle Couillin, Aurélie Gombault, Fabrice Trovero, Stéphan Chevalier, Pierre Besson, Lin-Hua Jiang, Sébastien Roger

**Affiliations:** 1 Inserm UMR1069-Nutrition, Growth and Cancer, University of Tours, 37032 Tours, France; lucie.brisson@univ-tours.fr (L.B.); julie.chamouton.pro@gmail.com (J.C.); stephane.chevalier@univ-tours.fr (S.C.); 2 EA4245-Transplantation, Immunology and Inflammation, University of Tours, 37032 Tours, France; stephanie.chadet@univ-tours.fr (S.C.); osbaldo.lopez@univ-tours.fr (O.L.-C.); bio_engineer1985@hotmail.com (B.J.); david.ternant@univ-tours.fr (D.T.); pierre.besson@univ-tours.fr (P.B.); l.h.jiang@leeds.ac.uk (L.-H.J.); 3 CNRS UPS44 TAAM, PHENOMIN, Centre d’Imagerie du Petit Animal, 45071 Orléans, France; stephanie.lerondel@cnrs-orleans.fr (S.L.); alain.lepape@univ-tours.fr (A.L.P.); 4 Laboratory of Molecular and Experimental Immunology and Neurogenetics, UMR 7355, CNRS, University of Orléans, 45071 Orléans, France; isabelle.couillin@cnrs-orleans.fr (I.C.); agombaul@cnrs-orleans.fr (A.G.); 5 Key-Obs: Preclinical CRO, 45100 Orléans, France; fabrice.trovero@key-obs.com; 6 School of Biomedical Sciences, Faculty of Biological Sciences, University of Leeds, Leeds LS2 9JT, UK; 7 Sino-UK Joint Laboratory of Brain Function and Injury and Department of Physiology and Pathophysiology, Xinxiang Medical University, Xinxiang 453003, China; 8 Institut Universitaire de France, 75005 Paris, France

**Keywords:** P2X7 receptor, cancer cell invasiveness, invadopodia, metastases, antagonists

## Abstract

The P2X7 receptor is an ATP-gated cation channel with a still ambiguous role in cancer progression, proposed to be either pro- or anti-cancerous, depending on the cancer or cell type in the tumour. Its role in mammary cancer progression is not yet defined. Here, we show that P2X7 receptor is functional in highly aggressive mammary cancer cells, and induces a change in cell morphology with fast F-actin reorganization and formation of filopodia, and promotes cancer cell invasiveness through both 2- and 3-dimensional extracellular matrices in vitro. Furthermore, P2X7 receptor sustains Cdc42 activity and the acquisition of a mesenchymal phenotype. In an immunocompetent mouse mammary cancer model, we reveal that the expression of P2X7 receptor in cancer cells, but not in the host mice, promotes tumour growth and metastasis development, which were reduced by treatment with specific P2X7 antagonists. Our results demonstrate that P2X7 receptor drives mammary tumour progression and represents a pertinent target for mammary cancer treatment.

## 1. Introduction

The P2X7 receptor (P2X7R) belongs to the family of ionotropic P2X receptors, which function as ATP-gated cation selective channels that are permeable to Na^+^, Ca^2+^ and K^+^ ions. However, P2X7R displays several functional and biological specificities differing largely from the other members of the P2X receptor family [1]. From a pharmacological point of view, a specific feature of P2X7R is to be 10–100 times less sensitive to its natural agonist ATP than all other P2X receptors, requiring millimolar concentrations to show measurable activation while all the others are activated by micromolar concentrations. In addition, P2X7R is about 10–30 times more sensitive to 2,3-O-(4-benzoylbenzoyl)-ATP (BzATP), a synthetic ATP analogue, than ATP [1]. A unique functional property of P2X7R is that upon prolonged or repeated agonist stimulation, it exhibits no desensitisation and instead striking facilitation or sensitization, a functional property characterized by an increase in the current response as well as an enhanced agonist sensitivity [2]. This unique functional property results in potentiation of downstream signalling pathways initiated by P2X7R.

While all other P2X receptors are mainly expressed in the nervous system, such as in neurons and glial cells, participating in the modulation of synaptic transmission, the P2X7R is mostly characterized by its predominant expression in immune cells and its critical role in immunity and inflammatory responses [3]. In the context of cancers, the P2X7R has attracted escalating attention over the past years. It has been reported that P2X7R is overexpressed in many different types of tumours, specifically in miscellaneous carcinomas [4], with the possible exception of cervical and endometrial cancers, in which it was intriguingly down-regulated [5,6]. Many studies provide evidence to show that P2X7R activity or activation protects cells from apoptosis [7], promotes cancer cell trophic activity and primary tumour growth [8,9] and stimulates cancer cell migration or invasion [10,11,12,13,14,15]. These studies thus favour a pro-cancerous role for P2X7R and propose that P2X7R antagonists are promising anticancer drugs [16]. In contrast, several other studies demonstrate that P2X7R induces cancer cell death upon ATP stimulation [17] or even enhances anti-tumour immune responses from the host organism, thus supporting the P2X7R to be anti-cancerous [18,19]. The role of P2X7R in cancer promotion appear to depend on multiple factors, including the type of cancers or tumours, the level of expression or activation of the receptor, the cell type considered (cancer cells vs. immune cells), the level of immune cell infiltration within the tumour and also the phase in the carcinogenic progression. Therefore, it remains unclear whether it would be beneficial, or detrimental, to antagonize P2X7R for anticancer purposes, and this needs to be addressed in specific cancer models.

In breast cancer, it has been shown that the P2X7R is overexpressed [20], fully functional and promotes cancer cell invasiveness, both in vitro and in an in vivo zebrafish model [12,21,22]. Moreover, P2X7R was demonstrated to induce the release of active proteolytic cysteine cathepsins [12], a finding that further suggests the involvement of P2X7R in the “mesenchymal mode” of invasion, in which cells remodel the extracellular matrix (ECM), by forming particular cellular structures that are enriched in F-actin and protrusive into the ECM called “invadopodia”, and using such proteolytic activity, generate their pathway [23].

In this study, we assessed the role of P2X7R in mammary cancer cells in determining the invasive properties, using both 2- and 3-dimensions models, more specifically, the invadopodial activity, and characterized the involvement of P2X7R in the acquisition of a mesenchymal phenotype in vitro. We also assessed the consequences of P2X7R expression in primary tumour growth and metastatic spreading in vivo. This study further aimed to clarify the relevance of pharmacological intervention using specific P2X7R antagonists in the treatment of mammary cancers. For this purpose, we developed an orthotopic, syngeneic and immunocompetent mammary cancer model in BALB/cJ mice, and studied the role of P2X7 receptor, expressed or not in 4T1 mammary cancer cells (using genetically engineered cells) or in the host organisms (wild-type *P2rx7^+^/^+^* versus knock-down *P2rx7^−^/^−^* mice). Our results unequivocally demonstrate that P2X7R is functionally expressed in mammary cancer cells and its activation promotes the acquisition of a mesenchymal phenotype and enhances invadopodial activity. Furthermore, we provide compelling evidence to indicate that the P2X7R expressed in mammary cancer cells but not in the host organism plays a key role in primary tumour growth and metastatic development, which are significantly attenuated by treatment with specific P2X7R antagonists. These findings support that the P2X7R in mammary cancer cells drives mammary tumour progression and represents a pertinent target for mammary cancer treatment.

## 2. Results

### 2.1. P2X7R Expression Promotes Mammary Cancer Cell Invasiveness

**Figure 1:**
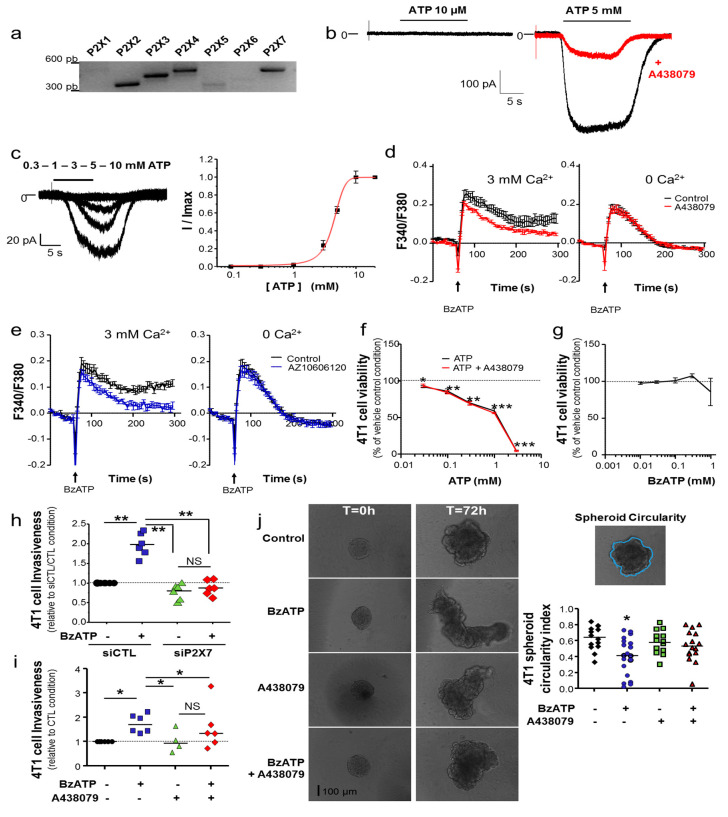
P2X7R is functional in 4T1 mouse mammary cancer cells and drives cell invasiveness. (**a**) RT-PCR analysis of P2X mRNA expression. (**b**) Representative whole-cell patch clamp recordings of ATP-induced currents. Membrane potential was held at −60 mV. While 10 s application of 10 µM ATP (left) evoked no detec current, application of 5 mM ATP produced a non-desensitizing current that was reduced by treatment with 10 µM A438079 (right). (**c**) Recordings of inward currents from one cell in response to 10 s applications of increasing concentrations of ATP (0.3, 1, 3, 5 and 10 mM) (left), and mean ATP dose-response curve, with currents expressed as a ratio of the maximum current obtained with 10 mM ATP (right). The solid line shows fit to Hill equation. (**d**) Changes in intracellular Ca^2+^ levels, indicated by the ratio of Fura2 fluorescence intensity induced by excitation at 340 and 380 nm (F_340/380_), by exposure to 300 µM BzATP in cells under control conditions (black traces) and cells treated with 10 µM A438079 (red traces) in extracellular Ca^2+^-containing solutions (left) or Ca^2+^-free solutions (right). These data are averaged from four independent experiments. There is statistically significant difference in BzATP-induced Ca^2+^ responses between control and A438079-treated cells in the presence of extracellular Ca^2+^ at *p* < 0.001, but no significant difference in the absence of extracellular Ca^2+^, using two-way ANOVA and Dunn’s Multiple Comparison post hoc test. (**e**) BzATP-induced Ca^2+^ responses in control cells (vehicle, black traces) and cells treated with 300 nM AZ10606120 (blue traces) in the presence of 3 mM extracellular Ca^2+^ (left) or in the absence of extracellular Ca^2+^ (right), using similar protocols as in d. These data are averaged from three independent experiments. There is a statistically significant difference in BzATP-induced Ca^2+^ responses between control and A438079-treated cells in the presence of 3 mM extracellular Ca^2+^ at *p* < 0.001, but no significant difference in the absence of extracellular Ca^2+^, using two-way ANOVA and Dunn’s Multiple Comparison post hoc test. (**f**) Cell viability after exposure to increasing concentrations of ATP (from 0.03 to 3 mM) for 24 h without or with treatment with 10 µM A438079, expressed as % of that under control conditions (vehicle). Results are from four independent experiments. ATP induced a statistically significant cell viability reduction compared to control conditions at * *p* < 0.05, ** *p* < 0.01 and *** *p* < 0.0001, but A438079 treatment had no significant effect on ATP-induced reduction in cell viability, using Student’s *t*-test. (**g**) Cell viability after exposure to increasing concentrations of BzATP (from 0.01 to 1 mM) for 24 h, expressed as % of that under control condition (vehicle). Results are from four independent experiments. BzATP induced no significant reduction in cell viability. (**h**), 4T1 cell invasiveness, assessed using trans-well invasion inserts after 24 h in the absence or presence of 300 µM BzATP, in cells transfected with control siRNA (siCTL) or P2X7R-specific siRNA (siP2X7). Results from six independent experiments expressed relative to the siCTL condition, in the absence of BzATP stimulation (CTL). ** indicates a statistically significant difference at *p* < 0.01 when comparing siCTL/BzATP to siCTL/CTL, using Mann-Whitney Rank Sum Test. NS stands for no statistical difference. (**i**) 4T1 2D-cell invasiveness was assessed over 24 h using trans-well invasion inserts in the absence or presence of BzATP (300 µM), and in the absence or presence of P2X7 antagonist A438079 (10 µM). Results are from 4–6 independent experiments and are expressed relatively to the control condition (CTL), in the absence of BzATP and of A438079. * indicates a statistically significant difference at *p* < 0.05 when comparing BzATP treatment to CTL, using Wilcoxon Signed Rank Test. (**j**) 4T1 3D-cell invasiveness was assessed over 72 h. The left image shows cancer cell spheroid morphology assessed before (T = 0 h) or after treatment (T = 72 h) in control condition (vehicle) or with BzATP (300 µM) in the absence or presence of A438079 (10 µM). Scale bar, 100 µm. On the right side is the summary of assessment of the spheroid circularity from 14–20 independent experiments. * indicates a statistically significant difference at *p* < 0.05 using Dunn’s Multiple Comparison Test.

In this study, we aimed at assessing the potential role of P2X7R in mammary cancer progression in an immunocompetent mouse model. Therefore, we investigated the expression and activity of P2X receptors in the 4T1 mammary cancer cell line, originating from the BALB/cJ mouse strain [24]. As shown in Figure 1a, 4T1 cells expressed mRNA transcripts for P2X2, P2X3, P2X4 and P2X7. A weak band can be visualized for P2X5. The functionality of these receptors at the plasma membrane of cancer cells were assessed using the patch-clamp recording technique. Stimulating the cells with 10 µM ATP, a concentration that would activate all P2X receptors with the exception of P2X7R, did not produce any measurable current. However, exposure to 5 mM ATP triggered inward, non-desensitizing, facilitating currents (Figure S1a) that were inhibited by treatment with A438079, a specific competitive P2X7 antagonist (Figure 1b). These results suggest that 4T1 cells mainly express functional P2X7R, while the other P2X receptors (P2X2 P2X3, P2X4 and P2X5) would be either not expressed at the protein level or not functional. To further characterize the ATP-induced currents, we constructed the ATP dose-current response relationship curve (Figure 1c) that yielded the concentration evoking 50% of the maximal current response (EC_50_) to be 4.3 ± 0.2 mM (*n* = 5–6 cells), consistent with the expression of the mouse P2X7R. We further used Fura2 fluorimetry to monitor the changes in intracellular Ca^2+^ levels in 4T1 cells in response to ATP (Figure S1b) or BzATP stimulation (Figure 1d). Both ATP and BzATP induced a biphasic increase in intracellular Ca^2+^ levels in cells incubated in extracellular Ca^2+^-containing solutions, with a transient component followed by a long-lasting one. The long-lasting Ca^2+^ increase was significantly reduced in the presence of A438079 or AZ10606120, a specific non-competitive P2X7R antagonist (Figure 1e), supporting P2X7R-mediated Ca^2+^ entry. In addition, the long-lasting, but not the transient, component was largely abolished in extracellular Ca^2+^-free solutions (Figure 1d,e, Figure S1b). Under these conditions, ATP/BzATP-induced intracellular Ca^2+^ increases were not affected by treatment with A438079 or AZ10606120, thus indicating that they are mediated by activation of G-protein coupled P2Y receptors. The P2Y11 receptor is known to be sensitive to both ATP and BzATP and coupled to intracellular Ca^2+^ release. The P2Y11 receptor was reported in cancer cells [25]. BzATP-induced intracellular Ca^2+^ increase in Ca^2+^-free solutions was attenuated by treatment with NF340, a P2Y11 selective antagonist (Figure S1c), in support of the role of the P2Y11 receptor in mediating ATP/BzATP-induced transient Ca^2+^ increase in 4T1 cells.

To explore the role of the P2X7R in cancer cell biology, we first assessed the effects of increasing ATP concentrations, from 0.03 to 3 mM, on 4T1 cell viability. As shown on Figure 1f, ATP significantly reduced the cell viability at all doses tested, which was not prevented by treatment with A438079. These results largely ruled out the participation of P2X7R in such an effect and suggest likely involvement of other P2 receptors or the receptors for adenosine, the major product of ATP hydrolysis, in the reduction of cell viability. This detrimental effect on cell viability was not observed when using BzATP as an agonist (Figure 1g). Therefore, to avoid any non-specific effect related to the activation of another purinergic signalling pathway, BzATP was the only agonist used in all the following experiments to stimulate the P2X7R and assess its role in the regulation of cancer cells. The participation of P2X7R in 4T1 cell invasiveness was first assessed in a 2-dimensional (2D) model using invasion filters covered with a film of Matrigel™, as an extracellular matrix (Figure 1h,i). In cells transfected with a null-target or control siRNA (siCTL), BzATP enhanced cancer cell invasiveness by a median factor of 1.95. Such a stimulatory effect was not observed in cells transfected with a siRNA specific for *P2rx7* gene (siP2X7) to reduce P2X7R expression (68.4 ± 1.6% (*n* = 6) assessed by RT-qPCR) (Figure 1h). Furthermore, BzATP-induced increase in invasiveness in non-transfected cells was prevented by treatment with A438079 (Figure 1i). We then assessed the effect of stimulating P2X7R on cancer cell invasiveness, using spheroids composed of 4T1 cells that were grown in a 3D matrix of Matrigel™ for 72 h (Figure 1j). 4T1 cells are very cohesive cells, tending to invade in a collective mode, rather than in an individual mode. Therefore, we measured the circularity index of spheroids, which is inversely correlated to the invasiveness, under different conditions. As shown in Figure 1j, exposure to BzATP reduced the spheroid circularity, indicating a gain in invasiveness, and BzATP-induced reduction of circularity was prevented by treatment with A438079. MDA-MB-435s human melanoma cancer cells were used as very aggressive human cancer cells, and P2X7R was shown to participate in determining the invasive capacities of these cells [11,12]. To compare mechanistic insights with those identified in mouse mammary 4T1 cancer cells, we performed similar experiments using spheroids composed of MDA-MB-435s cells. Under the same conditions, MDA-MB-435s cells tended to invade matrices in an individual cell mode (Figure S2a) and exposure to BzATP increased the maximal distance of matrix invasion, which was also prevented by treatment with A438079 (Figure S2b). Collectively, these results indicate that P2X7R activation promotes cancer cell invasiveness, regardless of the mode of invasion (collective vs. single cell invasion), in both 2D and 3D models.

### 2.2. P2X7R Participates in the Acquisition of a Mesenchymal Cancer Cell Phenotype

**Figure 2:**
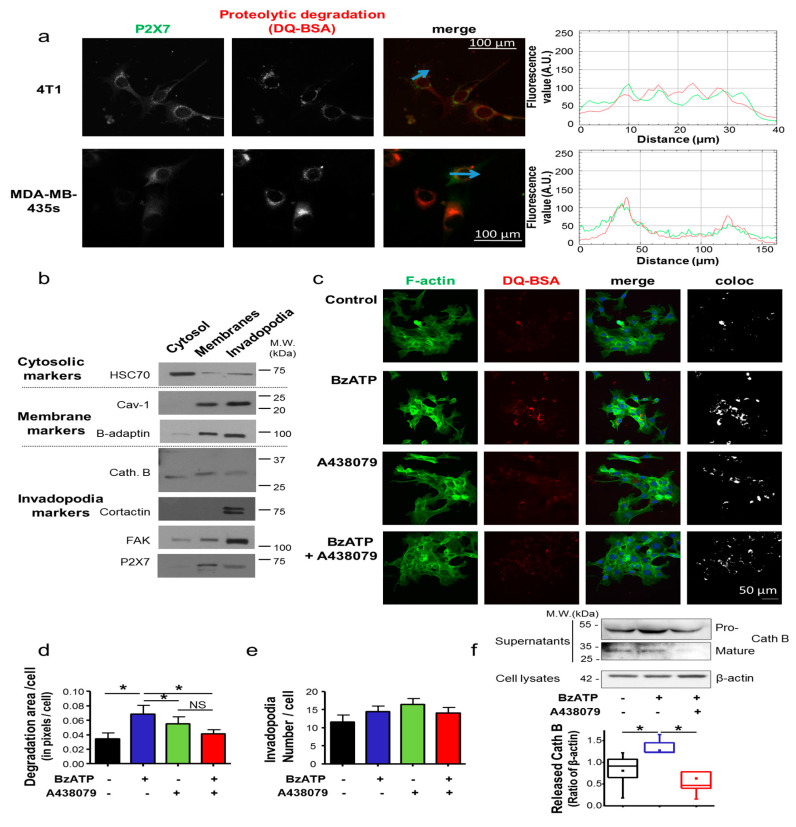
P2X7 receptor promotes invadopodial activity of extracellular matrix degradation. (**a**) 4T1 mouse mammary and MDA-MB-435s human cancer cells were grown on Matrigel™ containing DQ-BSA as a fluorogenic substrate for proteases, emitting red fluorescence when degraded. P2X7 receptor was immunodetected using a rabbit primary antibody against P2X7 protein and a secondary ant-rabbit IgG antibody conjugated to AF488. The right panel shows the fluorescence values for the two channels at the locations indicated by a blue arrow. Scale bar, 100 µm. (**b**) Invadopodia entrapped into a 2%-gelatin matrix were fractionated and separated from cytosol and membranes-enriched fractions. The quality of the fractions was assessed by western blotting after SDS-PAGE, using cytosolic (HSC70), membrane (caveolin-1, β-adaptin) and invadopodia (cathepsin B, cortactin, focal adhesion kinase) markers. P2X7 proteins were found to be present in the membrane fractions and enriched in the invadopodia fraction. This figure is representative of 5 independent experiments. The full image blots can be found in Figure S5. (**c**) The invadopodial activity was assessed as being F-actin foci (green labelling, phalloidin-488) co-localised with focused proteolytic activities (red labelling, DQ-BSA proteolysis) from 4T1 cells grown for 24 h on Matrigel™ containing DQ-BSA in control condition (vehicle), or stimulated with 300 µM BzATP, in the presence and absence of 10 µM A438079. “Coloc” indicates co-localization of degradative activity and F-actin foci and appears as white pixels. Scale bar, 50 µm. (**d**) The number of degradation areas per cell was counted in 20 images, from five independent experiments. * indicates a statistically significant difference at *p* < 0.05 using Dunn’s Multiple Comparison Test. NS stands for no statistical difference. (**e**) The number of invadopodia (identified as being F-actin foci colocalized with matrix degradation spots) was counted per cell in the same experiments as in d. There was no significant difference among the four treatment conditions. (**f**) Western blot analyses for cathepsin B (Cath B) in concentrated cell-free supernatants of 4T1 cells after 24 h treatment with BzATP (300 µM) in presence or not 10 µM A438079. Pro- and mature forms of Cath B were detected (top). β-actin was detected in corresponding cell lysates and was used as an internal control. Amounts of released mature forms of Cath B in supernatants of 4T1 cells in the three experimental conditions were analysed, from five independent experiments and expressed as a proportion of β-actin in corresponding cells. Box plots indicate median, first and third quartile and the mean is indicated as a square. * indicates a statistically significant difference at *p* < 0.05 using Dunn’s Multiple Comparison Test. The full image blots can be found in Figure S5.

Invadopodia are key structures of cancer cell invasiveness, involved in extracellular matrix (ECM) remodelling. Therefore, we analysed the ECM digestive activity of cancer cells grown on a planar matrix of Matrigel™ containing DQ-BSA as a fluorogenic substrate for proteases. Immunofluorescence imaging identified that P2X7R proteins were strongly co-localized with the matrix proteolysis (Figure 2a) in both mouse 4T1 (Pearson’s r = 0.64) and human MDA-MB-435s (Pearson’s r = 0.62) cancer cells. Since invadopodia are cancer cell structures that are protrusive into ECM, it is possible to isolate invadopodia-enriched fractions, from cytosolic or membrane fractions, coming from cancer cells grown on a thick matrix composed of gelatin [26]. Figure 2b shows that P2X7R proteins are present in the invadopodia fraction from 4T1 cells, characterised by the presence of the invadopodia markers, cortactin, focal adhesion kinase (FAK) and cathepsin B (Cath B). Similar results were obtained with human MDA-MB-435s cells (Figure S3a). Invadopodial activity can be characterised by focalized areas of ECM degradation co-localised with F-actin condensations. To assess a possible regulation of the invadopodial activity by P2X7R, 4T1 cells were grown on a planar matrix of Matrigel containing DQ-BSA, and F-actin was labelled (Figure 2c). Areas corresponding to the co-localization of F-actin condensation areas and focal spots of DQ-BSA proteolysis were identified and quantified per cell. Stimulating the cells with BzATP increased the surface of degradation spots, and this was prevented by treatment with A438079 (Figure 2d), whereas the number of invadopodial structures (degradation spots) per cell remained unchanged (Figure 2e). Similar results were obtained with human MDA-MB-435s cells (Figure S3b–d). These results strongly suggest that P2X7R activity enhances the proteolytic activity of individual invadopodia. In MDA-MB-435s human cancer cells, it is known that P2X7R activity facilitates release of multiple cathepsin isoforms including Cath B into the extracellular compartment [12]. We wondered whether P2X7R activation resulted in a similar effect in 4T1 cells. Indeed, exposure to BzATP increased the amount of mature Cath B in the supernatants of 4T1 cells by 2.12 ± 0.65 fold, and this was prevented by treatment with A438079 (Figure 2f).

**Figure 3:**
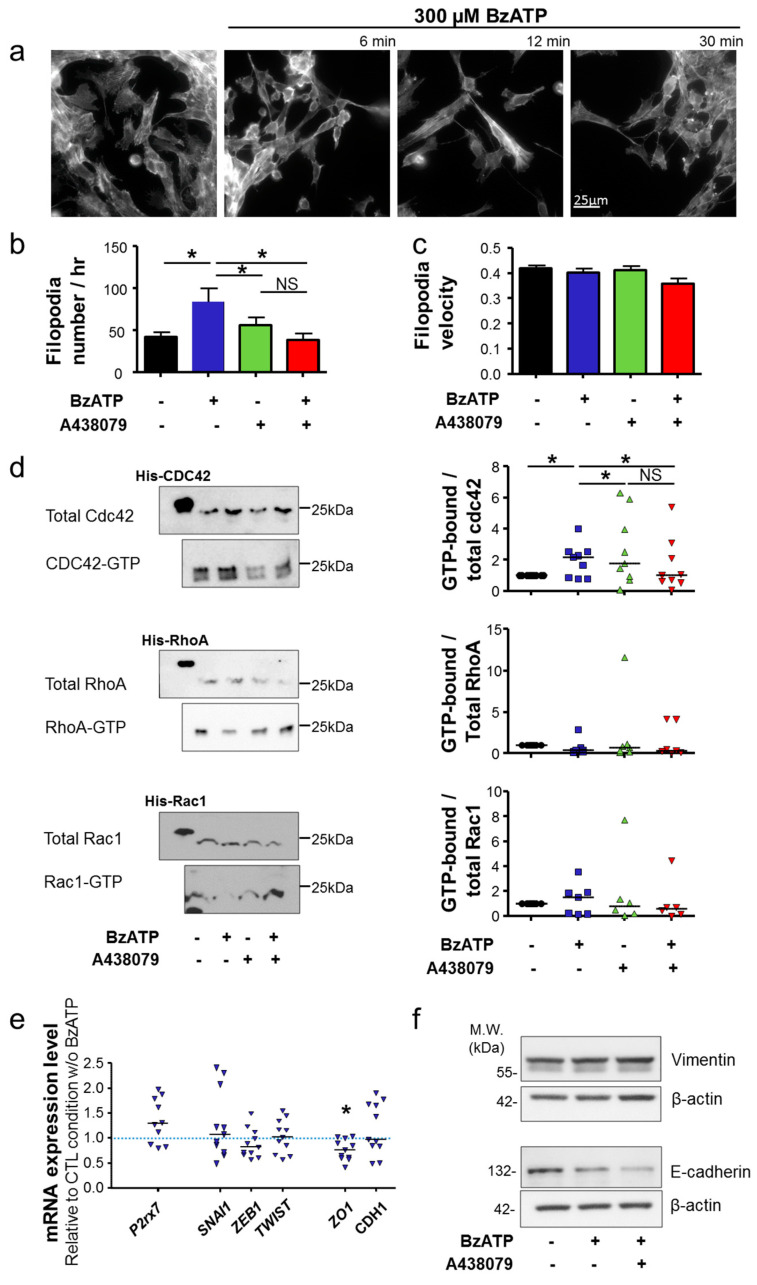
P2X7 receptor stimulation promotes the acquisition of a mesenchymal phenotype. (**a**) F-actin cytoskeleton was visualized using phalloidin-488 in 4T1 cells stimulated with 300 µM BzATP at the time indicated. Rapid morphological changes appear as rapidly as after 6 min stimulation. Scale bar, 25 µm. (**b**) The number of new filopodia per cell was counted per hour in cells transfected with the LifeAct plasmid and studied in the presence of 300 µM BzATP, in the presence or absence of 10 µM A438079. * indicates a statistically significant difference at *p* < 0.05 using Dunnett’s Multiple Comparison Test. (**c**) The velocity of filopodia to form and extend (in µm/min) was measured in the same experiments as in (**b**). There was no significant difference among the four experimental groups. (**d**) On the left side, representative western blots show total and active GTP-bound forms of Cdc42, RhoA, and Rac1 pulled down by GST-RBD in 4T1 cells in control conditions or stimulated for 30 min with 300 µM BzATP, in the presence and absence of 10 µM A438079. His-tagged proteins are used as positive controls for the quality of the pull-down experiment. On the right side, graphs show quantifications of GTP-bound RhoGTPases (active form), normalized to total protein level, and expressed relatively to that of the control condition (vehicle). Results are from 4–7 independent experiments. * indicates statistically significant difference at *p* < 0.05, using Mann-Whitney rank sum test. NS stands for no statistical difference. The full image blots can be found in Figure S7. (**e**) mRNA expression levels of *P2rx7*, *SNAI1*, *ZEB1*, *TWIST1*, *ZO1* and *CDH1* assessed by RT-qPCR in 4T1 cells stimulated by 300 µM BzATP for 24 h and expressed relatively to the GUSB reference gene and as ratios to the expression levels in control condition without BzATP (*n* = 9–11 independent experiments). SNAI1 was significantly higher, and ZO1 was significantly lower after BzATP treatment compared to control condition at * *p* < 0.05, using Mann-Whitney rank sum test. (**f**) representative western blots (from four independent experiments) show vimentin and E-cadherin expression in 4T1 cells in control conditions or stimulated for 24h with 300 µM BzATP, in the presence and absence of 10 µM A438079. There was no significant effect. The full image blots can be found in Figure S7.

The ECM digestive activity and the presence of invadopodia are specific characteristics of cancer cells showing a “mesenchymal phenotype” [27]. In this phenotype, engaged cells display an elongated fibroblast-like morphology, with a rear-to-front cell polarity, formation of lamellopodia at the leading edge and multiple filopodial structures around the cells. Filopodia are F-actin enriched thin structures allowing attachment to the matrix. As shown in Figure 3a, stimulation of 4T1 cells with BzATP induced a rapid change in cell morphology with a remodelling of F-actin cytoskeleton. Cancer cells appeared to be more elongated and the lamellopodia readily visualized. Time-lapse microscopy imaging (Videos S1–S4) also showed that exposure to BzATP increased the number of filopodial structures per cell, which was prevented by treatment with A438079 (Figure 3b). Of notice, treatment with A438079 alone was without effect on the number of filopodia. However, exposure to BzATP did not affect the velocity to form and extend filopodia (Figure 3c). It is known that the cytoskeleton remodelling and the acquisition of invasive phenotypes in cancer cells are orchestrated by members of the Rho-GTPase family, particularly, Cdc42 that has an important role in cancer cell invasion, acquisition of a mesenchymal phenotype, and formation of invadopodia [28]. Interestingly, exposure to BzATP increased the level of the active, GTP-bound form of Cdc42, with no effect on RhoA and Rac1, and such an effect on Cdc42 was abrogated by treatment with A438079 (Figure 3d). To further study the participation of P2X7R in the epithelial-to-mesenchymal transition (EMT), we assessed the effect of stimulating 4T1 cancer cells with BzATP on the expression of genes associated with the mesenchymal (*SNAI1, ZEB1, TWIST*) or the epithelial (*ZO1, CDH1*) phenotypes. Exposure to BzATP, while inducing no change in the expression of *SNAI1, ZEB1* and *TWIST*, repressed the expression of *ZO1* encoding for the epithelial tight junction protein Zonula occludens-1. Stimulating cells with BzATP had no effect on the expression of *CDH1*, encoding for E-cadherin (Figure 3e). Protein expressions of the mesenchymal marker vimentin and epithelial marker E-cadherin were also assessed. Vimentin protein level was not modified upon P2X7 stimulation. A trend for a decrease expression of E-cadherin was observed under BzATP treatment, but was not prevented by A438079, suggesting that there was no P2X7-attributable effect (Figure 3f).

Taken together, these results support the P2X7R as a strong promoter of invadopodial activity that participates in maintaining a mesenchymal aggressive phenotype in cancer cells without being critical to the induction of EMT.

### 2.3. P2X7R Enhances Mammary Tumour Growth and Metastasis Development and Is a Target for Anticancer Treatment

**Figure 4:**
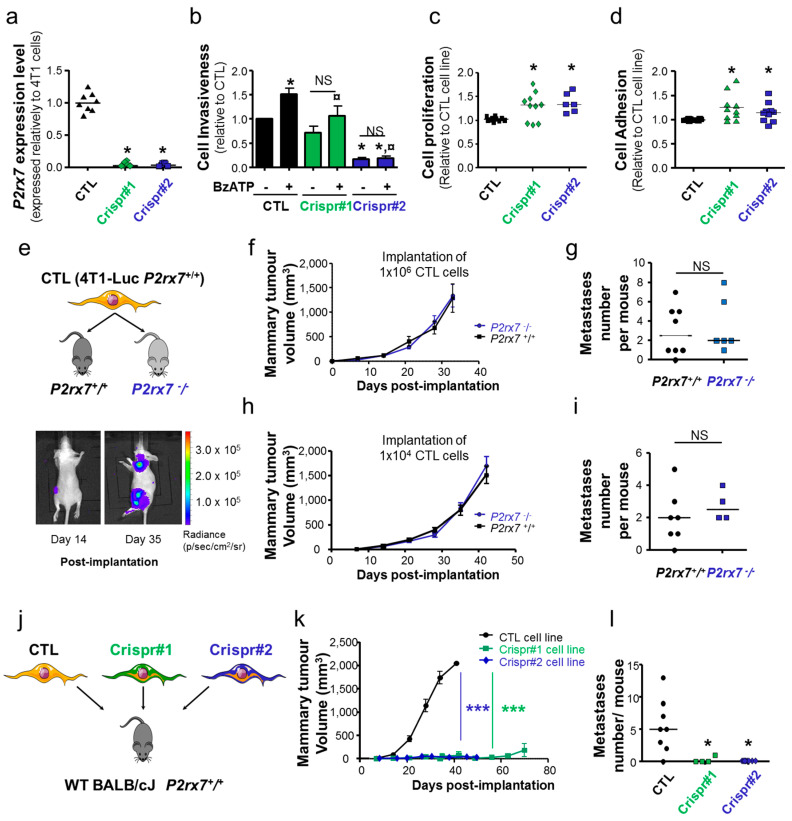
P2X7 receptor in mammary cancer cells enhances primary tumour growth and metastatic development in vivo. (**a**) *P2rx7* mRNA expression levels assessed by RT-qPCR in CTL, Crispr#1 and Crispr#2 cell lines, expressed relatively to 4T1 cells. * indicated a statistically significant difference from CTL cells at *p* < 0.05 using Mann-Whitney Rank sum test. (**b**) 2-D cancer cell invasiveness of CTL, Crispr#1 and Crispr#2 cell lines, in the absence or presence of 300 µM BzATP, expressed relatively to the CTL cell line without BzATP (*n* = 5–8 independent experiments). * indicates a statistically significant difference from CTL/no BzATP condition at *p* < 0.05, ^¤^ indicates a statistically significant difference from CTL/BzATP at *p* < 0.05, using Dunn’s Multiple Comparison Test. NS, stands for no statistically significant difference. (**c**) Cell proliferation over 4 days of CTL, Crispr#1 and Crispr#2 cell lines in the absence of 300 µM BzATP, expressed relatively to the CTL condition. Results are from 6–10 independent experiments. * indicates a statistically significant difference from CTL at *p* < 0.05 using Dunn’s Multiple Comparison Test. (**d**) Cell adhesion of CTL, Crispr#1 and Crispr#2 clones, expressed relatively to CTL. Results are from 10 independent experiments. * indicates a statistically significant difference from CTL at *p* < 0.05 using Dunn’s Multiple Comparison Test. (**e–i**) In vivo mouse experiments testing the effect of *P2rx7* gene expression in host mice on primary tumour growth and metastasis development, with two numbers of cancer cells implanted. (**e**) Top, cartoon describing the experimental procedure, in which CTL cells, derived from 4T1-Luc expressing *P2rx7* gene, were implanted to the fifth mammary fat pad of 6 weeks-old female wild-type BALB/cJ (*P2rx7^+^/^+^*) *or* P2X7 knock-out BALB/cJ *(P2rx7^−^/^−^*) mice. Bottom, representative bioluminescent images of a wild-type mouse, on 14 and 35 days after cell implantation, identifying primary tumours as well as metastatic foci. (**f**) Primary mammary tumour growth in wild-type BALB/cJ (*P2rx7^+^/^+^, n* = 8) *or* P2X7 knock-out BALB/cJ *(P2rx7^−^/^−^, n* = 8) mice as a function of time following the implantation of 1 × 10^6^ CTL cells on day 0. (**g**) Analysis of the number of metastases per mouse, identified at necropsy, in the experiment described in (**f**). NS stands for no statistical significant difference. (**h**) Primary mammary tumour growth in wild-type BALB/cJ (*P2rx7^+^/^+^, n* = 8) or P2X7 knock-out BALB/cJ (*P2rx7^−^/^−^, n* = 8) mice as a function of time following the implantation of 1 × 10^4^ CTL cells on day 0. (**i**) Analysis of the number of metastases per mouse, identified at necropsy, in the experiment described in (**h**). NS stands for no statistically significant difference. (**j–l**) In vivo mouse experiments assessing the role of *P2rx7* gene expression in mammary cancer cells on primary tumour growth and metastasis development. (**j**) Cartoon describing the experimental procedure, in which CTL (*P2rx7^+^/^+^),* Crispr#1 or Crispr#2 (both knock-down for the *P2rx7* expression) cells were implanted to the fifth mammary fat pad of 6 weeks-old female wild-type BALB/cJ (*P2rx7^+^/^+^*) at a density of 1 × 10^4^ CTL cells per mouse (8 mice per group). (**k**) Primary mammary tumour growth in wild-type BALB/cJ (*P2rx7^+^/^+^*) as a function of time following the implantation of CTL, Crispr#1 or Crispr#2 cells on day 0. *** indicates a statistically significant difference from the CTL group at *p* < 0.001 using two-way ANOVA. (**l**) Analysis of the number of metastases per mouse, in the three experimental groups, identified at necropsy, in the experiment described in (**j**). * indicates a statistically significant difference from the CTL group at *p* < 0.05 using Dunn’s Multiple Comparison test.

In order to assess the role of P2X7R in mammary tumour growth and metastatic progression, we genetically modified 4T1 cells for its expression, using the CRISPR/Cas9 gene-editing technique, generating three cell lines, two of which were named Crispr#1 and Crispr#2 with the *P2rx7* knocked-down with an efficacy of >95%*,* and one cell line named CTL expressing the *P2rx7* gene at a level similar to that in the parental 4T1 cells (Figure 4a). As a result, the Crispr#1 and Crispr#2 cell lines displayed a reduced invasiveness capacity compared to the CTL cell line and, furthermore, exposure to BzATP induced no potentiation of cell invasiveness (Figure 4b). Both Crispr#1 and Crispr#2 cell lines demonstrated a slight increase in proliferation (by a median factor of 1.32 and 1.33, respectively Figure 4c) and in cell adhesion (by a median factor of 1.19 and 1.15, respectively; Figure 4d) relative to CTL cells, under basal conditions, i.e., without agonist exposure. These three cell lines demonstrated a luciferase activity that was comparable to that of parental 4T1 cell line (Figure S4a), allowing their use for in vivo experiments. In order to decipher the role of P2X7R expressed by the cells of an organism hosting a mammary tumour, most importantly the immune cells, on primary tumour growth and metastasis development, we implanted CTL cells with two cell density (1 × 10^6^ or 1 × 10^4^ cells) in the fifth mammary fat-pad of either wild-type *(P2rx7*^+^/^+^*)* or knock-out *(P2rx7*^−^/^−^*)* BALB/cJ mice (Figure 4e). Surprisingly, regardless of the density of cancer cells implanted, there was no difference in either the mammary tumour growth (Figure 4f,h) or in the metastatic development (Figure 4g,i) between the *P2rx7*^+^/^+^ and *P2rx7*^−^/^−^ mice. These results indicate that in this model, the expression of P2X7R in host cells does not interfere with mammary tumour progression. Next, we assessed the role of the P2X7R expressed in cancer cells (Figure 4j). Genetic depletion of P2X7R expression in 4T1 cells strongly reduced mammary tumour growth in wild-type mice (Figure 4k) and also, most probably as a consequence of primary tumour reduction, markedly inhibited the development of metastatic foci (Figure 4l). The knock-down of *P2rx7* expression in Crispr#1 and Crispr#2 cells remained stable in the whole duration of the in vivo experiments (Figure S4b) as well as in primary tumours generated by these cells (Figure S4c). These results indicate that P2X7R in cancer cells (Figure 4c) rather than P2X7R in host animals (Figure 4f–i) promotes mammary tumour progression. Finally, we wondered whether treatment of mice bearing established mammary tumours with specific P2X7 antagonists would be effective to slow down the tumour growth.

**Figure 5:**
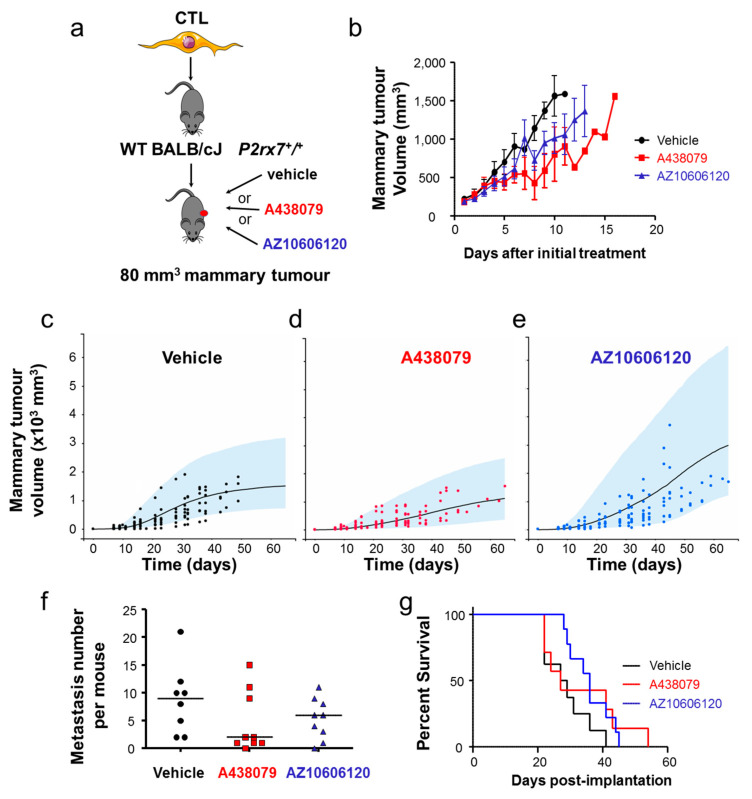
P2X7 antagonism slows mammary tumour growth. (**a**) In vivo mouse experiments assessing the effects of treatments with two P2X7 antagonist (A438079 or AZ10606120) on primary tumour growth, in which cancer cells and host organisms both express the *P2rx7* gene. (**b**) primary mammary tumour growth represented as a function of the duration of the treatment depending on antagonist treatments (A438079 or AZ10606120) compared to the injection of vehicle. (**c**–**e**) A Gompertz model was used to assess mammary tumour growth as a function of time for (**c**), vehicle group, (**d**), A438079 and (**e**), AZ10606120. Black, red and blue dots represent individual tumour volumes of these respective categories, blue areas are 90% model prediction intervals and lines are the median tumour growth. Estimated mean (inter-individual standard deviation) of model parameters were k_growth_ = 0.64 day-1 (39%), V_max_ = 1.620 mm^3^ (64%), EFF = 0.52 (82%) and γ = 0.17 (–). This analysis showed that tumour volume doubled in a mean time of 0.64 day, that the mean maximum tumour volume was 1.620 mm^3^ and that tumour growth was twice slower in the presence of A438079 or AZ10606120 treatment (*p* < 10^−10^) as compared to the vehicle group. There was no difference in the efficacy of these two treatments (*p* = 0.08). (**f**) Assessment of the number of metastases per mouse, in the three experimental groups; vehicle, A438079 and AZ10606120. There was no statistical difference. (**g**) Percent survival curve in the three experimental group indicating a significant prolonged survival of mice treated with AZ10606120 at *p* = 0.0464 using Gehan-Breslow-Wilcoxon Test.

Therefore, we injected intraperitoneally wild-type mice with A438079, AZ10606120 or vehicle as a control, when primary tumours reached a minimal size of 80 mm^3^ (Figure 5a). The results indicated a tendency for a reduction in tumour growth in mice treated with either P2X7 antagonist (Figure 5b). We further analysed these results using a Gompertz mathematical model applied to tumour growth (Figure 5c–e). Estimated mean (inter-individual standard deviation) of model parameters were k_growth_ = 0.64 day-1 (39%), V_max_ = 1.620 mm^3^ (64%), EFF = 0.52 (82%) and γ = 0.17 (–). Such analysis indicates that the tumour volume in the vehicle group doubled in a mean time of 0.64 day and the mean maximum tumour volume was 1.620 mm^3^ (Figure 5c). Treatment with A438079 (Figure 5d) or AZ10606120 (Figure 5e) led to a massive reduction in tumour growth by a factor of 2 (*p* < 10^−10^). There was no difference between treatments with these two antagonists (*p* = 0.08), even if a trend toward a better efficacy was observed for treatment with AZ10606120. In addition, V_max_ was significantly higher in the group treated with AZ10606120 (*p* = 0.0065), which may not be due to treatment itself, but rather due to between-subject variability. There was no statistically significant difference in the metastatic colonisation (Figure 5f). Treatment with AZ10606120 resulted in a significant increase in the survival rate (*p* = 0.0464, Figure 5g), and a trend for an improved survival rate was noticed in group treated with A438079. Taken together, these results consistently demonstrate that the P2X7R in mammary cancer cells rather than in host cells (i.e., mainly immune cells) is critical in driving mammary tumour growth and metastasis development and is a pertinent target for anticancer treatment.

## 3. Discussion

The acquisition of invasive capacities by cancer cells represents a critical mechanism in the cancer progression and metastasis that significantly impact the survival of cancer patients. Currently, there is no specific treatment for preventing cancer cell spreading from the primary tumour or preventing metastases appearance. At the cellular level, several modes of cancer cell migration and invasiveness have been described, both in in vitro and in vivo models. Among these are the “amoeboid” and the “mesenchymal” modes, that could be identified in both collective and individual cell invasions [29]. Even though these modes of invasion could be more characteristic of some cancer types or subtypes, cancer cells do not necessarily stay or are engaged in a specific one. The most aggressive cells often have the ability to switch from one mode to the other, depending on biological, physical and chemical conditions and constrains of the microenvironments [30]. Whatever the mode of invasion, being individual or collective, the capacity of degrading extracellular matrices that determines the dissemination rate of cancer cells critically depends on the activities of proteases, such as matrix metalloproteinases (MMP) and cathepsins. This capacity to invade extracellular matrices with a proteolytic degradation was initially described as being a feature of mesenchymal cancer cells. Mesenchymal cancer cells display an elongated fibroblast-like morphology, with a rear-to-front lamellopodial cell polarity, and harbour multiple cell-matrix adhesions, such as filopodial structures. The degradation of the ECM is recognized to be performed by invadosomal structures, which are F-actin-rich and protrusive into the ECM and responsible for its proteolysis through recruiting both membrane-associated and extracellularly-released soluble proteases [23,27].

Extracellular ATP is known to be important in the regulation of differentiation and activation of multiple cell types, under physiological conditions and also under pathological conditions. Over the past few years, ATP-induced purinergic signalling has attracted considerable attention in the field of oncology [4,31]. Indeed, ATP and other nucleotides have been demonstrated to be present in high concentrations in the tumour microenvironments, owing to the active release of ATP from cancer cells and to cell necrosis in the hypoxic halo of solid tumours [32,33]. When released in the extracellular compartment, ATP activates plasma membrane purinergic P2 receptors and downstream signalling pathways, which are well documented in different cell types in the tumour, such as cancer cells of course and also immune cells, fibroblasts and endothelial cells. Obviously, the consequences on tumour and disease progression could be drastically opposite, depending on the cell type considered, and therefore it is indispensable to develop a clear understanding of the diseases as a whole. The P2X7R is a very intriguing receptor, which has been demonstrated to be upregulated in multiple cancer types [4], but its clear involvement in carcinogenesis or cancer progression is still debated. Because the over-stimulation of P2X7R, either in dose or in duration, is known to induce cell death [34], several studies postulated that it might be non-functional in proliferating cancer cells [20,35]. It was even proposed that stimulating its activity in cancer cells would induce their death and could represent therapies for treating cancers [36]. However, multiple studies demonstrated that the P2X7R is expressed and functional in cancer cells and that its basal activity or its external stimulation with biologically relevant concentrations of ATP support cancer cell growth, migration and invasion [12,13,37], and tumour growth [9,14]. In these models, pharmacological antagonism of P2X7R appeared to be effective in reducing cancer progression, suggesting that targeting P2X7R could represents a novel opportunity for anticancer treatments.

It is now clearly established that the ATP concentration can reach mM in the necrotic areas of solid tumours. Such massive necrosis, which provides the large amount of ATP required to activate the P2X7R, mainly occurs in the inner core of the tumour (e.g., being ischemic and hypoxic) with a decreasing gradient toward the outer part of the tumour. The outer part is involved in local invasion and subsequent metastasis. Under such conditions, it is likely that excessive concentration of ATP in the necrotic core induces cancer cell death, while a moderate concentration of ATP present at the edge of the growing tumours stimulates invasion, through activating the P2X7R in cancer cells, as proposed in the “run or die” hypothesis [16]. Also, it is not well understood but it may be important for further studies to assess the P2X7R expression within the tumour and compare the expression level in the inner core and the outer layer of the tumour.

On the other hand, the important role of P2X7R in the immune responses is well established [3]. Its expression and activity in cells from the host organism of a tumour might interfere with tumour progression. Importantly, its role in tumour-associated dendritic cells for the presentation of tumour antigen to CD4^+^ lymphocytes, and its role in anti-tumour immune responses were demonstrated [18,38]. Therefore, it was questioned whether P2X7R had a real role in cancer progression, as well as whether using P2X7 antagonists was pertinent and useful for anticancer treatment. In a very nice study, Adinolfi and collaborators [39] demonstrated that, when expressed in mouse melanoma or colon cancer cells subcutaneously inoculated to P2X7R-deficient mice, P2X7R strongly promoted tumour growth and metastatic dissemination. However, both primary tumour growth and metastases were reduced in P2X7R-expressing wild-type mice because of a P2X7R-dependent anti-tumour immune response [39]. Another study, performed in the context of colon cancer, also supports an important role for P2X7R in anti-tumour immune response. In this study, Hofman and collaborators [40] showed that pharmacological antagonism or genetic silencing of the P2X7R altered immune cell infiltration and increased tumour incidence in a mouse model of colitis. Therefore, from these studies it appeared that the use of P2X7 antagonists for anti-cancer treatment might be deleterious and could lead to opposite tumour-promoting effects. Melanoma and colon cancer development and progression are known to be highly dependent on the immune and inflammatory system [41,42]. For other cancers such as mammary cancer, the question is still open, and the relative participation of P2X7R expressed by cancer cells or host cells might be different and should be specifically assessed. Breast cancer is the primary cause of death by cancer in women worldwide and patients mostly die because of metastases appearance and development [43]. Breast tumours are also highly heterogeneous and there are four major molecular subtypes differing for their prognostic and treatments: Luminal A, Luminal B, HER2-enriched and Triple negative/basal-like. Triple negative/basal-like tumours are often very aggressive and have a poorer prognosis compared to the ER-positive subtypes (luminal A and luminal B tumours) [44].

In this study, we have examined 4T1 mouse mammary cancer cells, which are a model of triple-negative mammary tumours. We demonstrated that the P2X7R is functional in 4T1 cancer cells and that its activation with natural (ATP) or synthetic (BzATP) agonists induces typical facilitating inward currents and increases in intracellular Ca^2+^ levels. Its stimulation did not seem to induce cell death, even though the genetic silencing of the *P2rx7* gene led to a slight increase in the proliferation rate, and instead importantly enhanced cell invasiveness under 2D and 3D conditions. These effects were similarly prevented both by treatment with pharmacological antagonists and by genetic depletion, indicating that not only the P2X7R expression but also its activity is important in promoting cell invasiveness. These results are in agreement with previous reports performed with human cancer cells [11,12,13,15,22,45]. While mechanistic determinants for such a pro-invasive role were not clearly delineated, we have identified for the first time in this study the P2X7R expression in invadopodial structures involved in ECM degradation. More specifically, the P2X7R expression enhanced the ECM-degradative activity of invadopodia, most likely through stimulating release of proteolytic enzymes [12] such as Cath B (Figure 2f), rather than formation of these structures *per se*. Activation of the P2X7R also importantly modified cancer cell morphology and triggered the acquisition of a more aggressive phenotype, characterized by activation of Cdc42 Rho-GTPase, remodelling of F-actin, elongation of cells and formation of filopodia. Taken together, the results presented in this study strongly suggest the involvement of P2X7R in promoting the acquisition of a mesenchymal invasive phenotype in mammary cancer cells. Activation of P2X7R led to a reduction in the expression of *ZO-1*, which is important in maintaining tight junction function and epithelial polarity. However, in our model, activation of the P2X7R appears not important in inducing EMT. Indeed, there was no significant change in the expression of EMT-promoting transcription factors, Zeb1, Snail1 or Twist, and the expression of the gene encoding for E-cadherin was not modified. The protein expression of the mesenchymal marker vimentin was not modified either. These findings are different from what are reported in recent studies examining other cancer types. In prostate cancer cells, P2X7R was shown to promote invasiveness and metastatic properties and silencing its expression attenuated ATP- or BzATP-induced changes in the expression of EMT-related genes, Snail, E-cadherin and Claudin-1 [15]. In osteosarcoma cells, stimulation of P2X7R reduced the expression of E-cadherin, and induced the expression of Snail, vimentin and fibronectin. These effects were prevented by pharmacologically antagonizing the P2X7R or knocking down its expression [45]. However, these differences might depend on the cell types studied and on their level of transition. Indeed, it was reported that murine 4T1 mammary cancer cells, which are highly invasive and metastatic, do not strictly exhibit the genotypic and phenotypic properties of EMT [46]. It was postulated that other processes may govern the metastatic capability of these cells. Furthermore, it is know that EMT is not an all-or-nothing phenomenon, and some cancer cells display intermediate or partial transition states that have been identified to be highly aggressive [47].

Here, we investigated the role of P2X7R, whether it was expressed by cancer cells or by host cells, in mammary cancer progression. By using *P2rx7*^+^/^+^ and *P2rx7*^−^/^−^ mice, we demonstrated that, in the syngeneic and orthotopic model of mammary cancer, there is no participation of the P2X7R in the host organism in either anti- or pro-tumour activities. Because the level of the anti-tumour response of the host organism might depend on the size of the primary tumour, we inoculated two different numbers of mammary cancer cells (low density of 1 × 10^4^ or high density of 1 × 10^6^ cells per inoculation site) but no difference was observed. Such a finding is in apparent contradiction to what were reported by previous studies [39,40], but may be due to the specific mammary gland microenvironment [48], as compared to the subcutaneous or colic environments, or to specific anti-immune responses induced by 4T1 cells. Such possibilities should be investigated in further studies. However, in stark contrast, the expression and activity of P2X7R in mammary cancer cells had a predominant effect in mammary tumour growth and metastasis development. Loss of the *P2rx7* expression in two cell lines derived from 4T1 cells led to a significant delay in primary tumour growth, while the cells demonstrated a slight increase in the proliferation rate compared to the *P2rx7^+^/^+^* cells. The consistent results using two different *P2rx7*-knocked-out clones rule out possible off-targets of the CRISPR/Cas9 technique used and demonstrate the critical involvement of cancer cell invasiveness in primary tumour growth. It is interesting to notice that while knocking out the expression of P2X7R in 4T1 cells slightly increased their proliferation rate in vitro, it completely abolished the growth of the primary mammary tumour in vivo. One possible explanation is that, by promoting the proteolytic degradation of the ECM and local invasion, P2X7R importantly supports primary tumour growth. We also demonstrated a clear reduction of metastatic colonization of organs when cancer cells did not express the *P2rx7* gene. However, it remains unclear whether this effect is specific or due to the important delay in primary tumour growth. Furthermore, we demonstrated that treatment with specific P2X7 antagonists with different modes of action, one being competitive (A438079) and the other being non-competitive (AZ10606120), had similar effects in reducing the tumour growth in wild-type mice.

## 4. Materials and Methods

### 4.1. Agonists, Antagonists, Salts and Chemicals

Adenosine 5′-triphosphate (ATP) disodium salt and 2′-(3′)-O-(4-benzoylbenzoyl) adenosine 5′-triphosphate (BzATP) triethylammonium salt were purchased from Sigma-Aldrich (St. Quentin Fallavier, France) and prepared in PBS without Ca^2+^. A438079, AZ10606120 and NF340 were purchased from Tocris Bio-Techne (Noyal Châtillon sur Seiche, France) and prepared in saline solution. All salts and pH buffers for electrophysiological solutions were purchased from Sigma-Aldrich. Fluorescent probes (Phalloidin DyLight^®^ 488, DQ™-BSA and Fura2-AM) were all purchased from Invitrogen (Thermo Fisher Scientific, Villebon-sur-Yvette, France).

### 4.2. Cells and Cell Culture

Human melanoma cancer cell line MDA-MB-435s-luc (thereafter called “MDA-MB-435s”) was constructed as previously described [49]. Murine mammary cancer cell line 4T1 from the Balb/c strain was purchased from LGC Standards (Molsheim, France), and a stable 4T1-luc cell line expressing the luciferase gene (thereafter called “4T1 cells”) was obtained by transduction with lentiviral vectors containing the luciferase gene and blasticidin resistance gene for selection (GIGA Viral Vectors, Liege, Belgium). stable 4T1 cell lines knocked-out for the expression of the *P2rx7* gene were obtained using the CRISPR/Cas9 technique by transfection with the *P2rx7* Double Nickase Plasmid (Santa Cruz Biotechnology, CliniSciences, Nanterre, France). Clonal selection was performed using 2 µg/mL puromycin. Two clones have been kept for this study, called “Crispr#1” and “Crispr#2”. A null-target Double Nickase Plasmid was also used to transfect 4T1 cells, leading to the selection of a control cell line, thereafter called “CTL” cell line. Efficiency of the CRISPR-mediated knock-down was assessed by RT-qPCR and invasion assays, and stability of clones was followed for a minimal duration of 6 weeks. Selected clones displayed similar levels of luciferase activity.

MDA-MB-435s cells were grown in Dulbecco’s modified Eagle’s medium (DMEM) supplemented with 5% foetal calf serum (FCS). All 4T1-derived cells were cultured in RPMI medium supplemented with 10% FCS. Cells were grown at 37 °C in a humidified 5% CO_2_ incubator. Mycoplasma contamination tests were performed routinely (MycoAlert™ Mycoplasma Detection Kit, Lonza, Thermo Fisher Scientific, Villebon-sur-Yvette, France).

### 4.3. Small Interfering RNA Transfection

4T1 mammary cancer cells were transfected with siRNA directed against mouse *P2rx7* mRNA (siP2X7) or scrambled siRNA as a control (siCTL), both of which were purchased from Tebu-Bio (Le Perray-en-Yvelines, France). Cells were transfected with 20 nM siRNA by using Lipofectamine RNAi max (Invitrogen). Experiments were performed 24 h after transfection and efficacy of silencing was assessed by RT-qPCR.

### 4.4. RNA Extraction, Reverse Transcription and Polymerase Chain Reaction

For conventional RT-PCR, total RNA was extracted using NucleoSpin^®^ RNA II kit (Macherey Nagel EURL, Hoerdt, France) and reverse transcribed with PrimeScript™ RT Reagent (Ozyme, Saint-Cyr-l’École, France). PCR was performed with GoTaq^®^ Flexi DNA Polymerase (Promega, Charbonnières-les-Bains, France) according to manufacturer’s recommendations. PCR products were loaded on 2% agarose gels and visualised after UV excitation.

For quantitative PCR (qPCR), total RNA was extracted using TRIzol™ Reagent (Invitrogen), and reverse-transcribed with the PrimeScript™ RT Reagent Kit (Ozyme, France). PCR were performed using SYBR qPCR Premix Ex Taq (Ozyme, France) and CFX CONNECT (Bio-rad, Marnes-la-Coquette, France). All primers sequences are described in Table 1.

### 4.5. Electrophysiology

Electrophysiological recordings of ATP-induced ionic currents were performed in the whole- cell configuration of the patch clamp technique. Patch pipettes were pulled from borosilicate glass (World Precision Instruments, Hitchin, UK) to a resistance of 4–6 MΩ. Currents were recorded under voltage-clamp mode using an Axopatch 200B amplifier (Axon Instrument, Molecular Devices, San Jose, CA, USA) and analogical signals were filtered at 10 kHz and digitized using a 1322A Digidata converter (Molecular Devices, San Jose, CA, USA). Cell capacitance and series resistance were electronically compensated. Membrane potential was held at −60 mV. Experiments were performed at room temperature (20–25 °C) in extracellular physiological saline solution (PSS in mM: 147 NaCl, 10 N-2-hydroxyethylpiperazine-N′-2ethansulphonic acid (HEPES), 13 D-glucose, 2 KCl, 2 CaCl_2_ and 1 MgCl_2_) and pipettes were filled with intracellular saline solution (in mM: 147 NaCl, 10 HEPES and 10 ethylene glycol-bis-(2-aminoethyl ether)-N, N, N′, N′-tetraacetic acid (EGTA) with osmolarity and pH values of 295–315 mOsm and 7.3, respectively). ATP was externally applied using a RSC160 fast-flow delivery system (BioLogic Science Instruments, Seyssinet-Pariset, France) for 10 s, at the concentrations indicated in the Figure 1 legend. P2X7 antagonist A438079 was perfused into the bath for 2 min before recording its effect in the presence of 5 mM ATP. ATP concentration-response curves were obtained by first obtaining a maximum response to 10 mM and then by applying decreasing concentrations of ATP and the results were plotted using Origin Pro 2015 software (Microcal Software Inc., Northampton, MA, USA). EC_50_ was derived by fitting the data to Hill equation provided by the software.

### 4.6. Intracellular Ca^2+^ Measurement

ATP- and BzATP-induced increases in intracellular Ca^2+^ levels were measured using ratiometric fluorescent probe Fura2-AM and a Flexstation3 microplate reader (Molecular Devices) as previously described [25]. Cells were incubated with 1 µM Fura2-AM for 45 min in OptiMEM medium at 37 °C prior to conduct recordings. Cells were washed and then pre-incubated in the extracellular saline solution (in mM: 147 NaCl, 2 KCl, 1 MgCl_2_, 10 HEPES and 13 D-glucose, pH 7.4) with and without 3 mM CaCl_2_. F340/F380 defined the ratio of the fluorescence intensity excited alternatively at 340 nm and 380 nm and emitted at 510 nm and was used to indicate the free intracellular Ca^2+^ concentration. 3 mM ATP or 0.3 mM BzATP was applied to cells after the baseline was established. Antagonists, A438079 (10 µM) or AZ10606120 (300 nM) were added 5 min before application of agonist.

### 4.7. Cell Viability

The effects of exposure to ATP and BzATP at doses indicated in the figure legends for 24 h on 4T1 cell viability were evaluated by using the tetrazolium salt assay (MTT) as previously described [12]. Briefly, cells were treated with increasing doses of ATP and BzATP for 24 h and cell viability was measured after incubation with MTT for 40 min at 37 °C.

### 4.8. Cell Adhesion

To assess 4T1 cancer cell adhesion, 2 × 10^4^ cells were seeded in their normal culture medium in wells of a 96-well plate and placed in the incubator for 30 min at 37 °C and 5% CO_2_. After one wash in PBS, the number of adherent cells was evaluated in wells using the MTT assay.

### 4.9. Two- and Three-Dimensions In Vitro Invasion Assays

2-D cancer cell invasiveness was measured as previously described [12] using 8 µm pore-size polyethylene terephtalate membrane inserts covered with Matrigel™ matrix (Becton Dickinson, Le Pont de Claix, France). Cells at the lower surface of the insert were stained with DAPI and nuclei were counted after collecting pictures with a Nikon TI-S microscope (Nikon S.A.S., Champigny-sur-Marne, France). Results were normalized to the control condition in the absence of agonist or antagonist.

For 3D invasiveness assays, cancer cell spheroids were used. To do so, 500 4T1 cells or 2000 MDA-MB-435s cells were seeded in wells of ultra-low attachment 96-well plates (Corning, Boulogne-Billancourt, France). After allowing spheroid formation at 37 °C and 5% CO_2_ in the incubator for 24 h for 4T1 and 48 h for MDA-MB-435s, 4 mg/mL Matrigel™ was added to the wells, in the absence or presence of 0.3 mM BzATP, and 10 µM A438079. 3D-Matrigel invasion by spheroids was followed every hour using a Nikon TI-S microscope. Invasion distance and spheroid circularity were measured and calculated using ImageJ version 1.48.

### 4.10. Invadopodia Activity Assay

Invadopodia activity was assessed by culturing cells for 24 h on the top of a layer of Matrigel™ (4 mg/mL) matrix containing 50 µg/mL of DQ-BSA in LabTeck™ (Palaiseau, France) chambers, as previously reported [26]. Briefly, cells were fixed in 4% paraformaldehyde for 15 min, permeabilized using 0.02% saponin for 20 min and incubated with 3% bovine serum albumin (BSA) for 30 min. F-actin was stained with 1.5 units/mL Phalloidin DyLight 488 (Invitrogen). Slides were mounted using ProLong^®^ Gold Antifade Mountant with DAPI (Invitrogen). Epifluorescence microscopy was performed with a Nikon TI-S (Nikon). Co-localized pixels for DQ-BSA and Phalloidin-488 were obtained using NIS-Element (Nikon) and fluorescence intensity, as well as the number of invadopodia, was quantified with ImageJ.

### 4.11. Invadopodia Fractionation

Invadopodia, which were embedded in the gelatin matrix, were separated from cellular bodies using the previously described protocols [26]. Briefly, cells were grown on the top of a 2%-gelatin matrix. Cellular bodies were removed using osmotic shock and further fractionated to isolate a “cytosolic” and an “all membranes” fractions. Proteins from invadopodia were solubilised in a lysis buffer containing 0.1% NP-40 and 1 mM DTT and separated from gelatin by centrifugation at 17,000× *g* for 30 min. Proteins in each fraction were separated according to standard SDS-PAGE protocols on 8% and 12% polyacrylamide gels and then transferred on PVDF membrane. Primary antibodies used were: mouse anti-HSC70 (sc-7298 Santa Cruz Biotechnology, Inc., Heidelberg, Germany), rabbit anti-caveolin 1 (sc-894 Santa Cruz Biotechnology, Inc., Germany), rabbit anti-β-adaptin (610382 BD Biosciences, Le Pont de Claix, France), cathepsin B (20-CR71 Fitzgerald, Acton, MA, USA), cortactin (05-180 Millipore, Guyancourt, France), FAK (036SC-558 Santa Cruz Biotechnology, Inc., Heidelberg, Germany), P2X7 (APR-008 Alomone Labs Ltd., Jerusalem, Israel). Secondary HRP-conjugated antibodies were: goat anti-mouse (Santa Cruz Biotechnology, Inc., Heidelberg, Germany), goat anti-rabbit (Jackson Immunoresearch Interchim, Montluçon, France), rabbit anti-β-actin-HRP (Santa Cruz Biotechnology, Inc., Heidelberg, Germany). Densitometric analyses were performed using ImageJ. Full uncropped blots are shown in Figures S5 and S6.

### 4.12. Epifluorescence Experiments

Cells were grown for 24 h in LabTeck™ chambers on a layer of Matrigel matrix (4 mg/mL) containing 50 µg/mL of DQ-BSA. Cells were fixed in 4% paraformaldehyde for 15 min and then incubated with 3% BSA for 30 min. P2X7 was immunodetected using a primary antibody (APR-008, Alomone Labs Ltd., Israel) and a secondary anti-rabbit IgG AlexaFluor488 (Invitrogen, France). For actin cytoskeleton analysis, cells were grown on glass coverslips until 40% confluency and then serum was reduced to 1% for 24 h and 0% for 24 h. Cells were treated with BzATP for 2, 4, 6, 12, 30 min and 24 h. Cells were fixed in 4% paraformaldehyde for 15 min, permeabilized with 0.1% triton-X-100 for 5 min, incubated with 3% BSA for 30 min and stained with 1.5 units/mL Phalloidin DyLight^®^ 488 (Invitrogen, France) for 1 h. Slides were mounted using ProLong^®^ Gold Antifade Mountant with DAPI (Invitrogen, France). For the analysis of filopodia, cells were transfected with Ibidi^®^ LifeAct plasmid (Generously provided by Laurent Counillon, CNRS UMR7370, University of Nice-Sophia Antipolis, Nice, France) using TransIT^®^-2020 (Mirus Euromedex, Souffelweyersheim, France). Epifluorescence and time-lapse microscopy was performed with a Nikon TI-S (France) (see Videos S1–4). Numbers of filopodia formed per hour and filopodia velocity were quantified using the Adapt plugin for ImageJ.

### 4.13. RhoGTPases Pull-Down Assays

The activity of RhoA, Rac1 and Cdc42 was assessed using pull-down assays according to the manufacturer’s protocols (Cat BK030 RhoA/ Rac1/Cdc42 Activation Assay Combo Biochem Kit, Cytoskeleton, Tebu-Bio, France) as previously described [50]. Briefly, cells were grown until 40% confluency in 75 cm^2^ flasks in normal growing medium, and serum was reduced to 1% for 24 h and 0% for 24 h. After treatment with 0.3 mM BzATP with or without 10 µM A438079 for 30 min in culture medium without serum, cells were lysed and samples were snap-frozen in liquid nitrogen. For each condition, 500 µg total lysates were used for pull-down assay according to the manufacturer’s instructions and then used for western blotting (Full uncropped blots are shown in Figure S7).

### 4.14. Western-Blotting Experiments

Cells were washed twice with PBS and lysed in presence of a lysis buffer (50 mM Tris, pH 7, 100 mM NaCl, 5 mM MgCl2, 10% glycerol, 1 mM EDTA), containing 1% Triton-X-100 and protease inhibitors (Sigma-Aldrich, France). Cell lysates were cleared by centrifugation at 16,000× *g* for 10 min. Total protein concentrations were determined using the Pierce^®^ BCA Protein Assay Kit (Thermofisher Scientific, Villebon-sur-Yvette, France). Protein sample buffer was added and samples were boiled at 100 °C for 3 min. Samples were loaded (20 μg of total proteins for cell lysates and 6 μL of concentrated supernatants) and electrophoretically separated on 10% or 12% polyacrylamide gels and then transferred to polyvinylidene difluoride membranes. Vimentin was detected using a primary anti-vimentin rabbit antibody (1/1000, clone D21H3, Cell Signaling Technology, Danvers, MA, USA) and a secondary horseradish peroxidase (HRP)-conjugated goat anti-rabbit IgG antibody at 1:2000 (TEBU-BIO, Le Perray-en-Yvelines, France). E-cadherin was detected using a monoclonal mouse primary antibody (1/1000, HECD-1, 13-1700 ThermoFisher Scientific) and a secondary HRP-conjugated anti-mouse-IgG secondary antibody at 1:2000 (TEBU-BIO). β-actin was used as a sample loading control using anti-β-actin-HRP primary antibody at 1:1000 (C4, Santa Cruz Biotechnology ref sc-47778). Western blot experiments for the detection of both pro and mature forms of cathepsin B were performed from 4T1 concentrated supernatants. Cells were grown to 80% confluence in a 6-well plate. After 24 h treatment (BzATP ± A438079) in Optimem medium (Invitrogen, France), cell supernatants were taken and concentrated using 10,000 MWCO filters (Merck Millipore, Molsheim, France). Cathepsin B proteins were visualized using polyclonal rabbit anti-human cathepsin B primary antibodies (1/150, Fitzgerald) and a goat anti-rabbit horseradish peroxidase (HRP)-conjugated secondary antibody (1/5000).Proteins were revealed using electrochemiluminescence-plus kit (Pierce ECL Western Blotting Substrate, ThermoFisher Scientific, France) and captured on a PXi acquisitions system (SYNGENE, Cambridge, UK). Densitometry analysis of protein bands was performed using the Gel Tool from Fiji software (Scientific image analysis software available at https://fiji.sc). Full uncropped blots are shown in Figures S5 and S6.

### 4.15. In Vivo Mammary Cancer Model and Experiments

All experiments have been approved by the Comité d’éthique du Centre-Val de Loire (Comité d’éthique en expérimentation animale Campus CNRS d’Orléans n°3, Ref 005377.01 Apafis #12960) and were performed in accordance with the European Ethics rules. All animals were bred and housed in isolated ventilated cages at the CNRS UPS44—TAAM-CIPA (CNRS Campus, Orléans, France), in controlled conditions with a 12 h light/dark cycle at 22 °C, and food and water *ad libitum*. We developed a syngeneic and orthotopic mouse mammary cancer model in female BALB/cJ immunocompetent mice. To do so, 4T1-luciferase-expressing mouse mammary cancer cells were injected into the fifth mammary fat pad of 6 weeks-old mice. The luciferase activity was mainly used to follow secondary tumour appearance and growth in vivo, subsequent to D-luciferin (150 mg/kg) intraperitoneal injection and bioluminescent imaging (IVIS Lumina II, Perkin Elmer, Villebon-sur-Yvette, France). Primary tumour volume (mm^3^) and growth over time were most effectively measured with a calliper, twice a week, and calculated as (L × l^2^)/2 (in mm). Metastases were counted macroscopically at the completion of studies, during autopsies. Animal weight was measured once a week.

In experiments comparing the role of the number of injected cells on primary tumour growth, in mice expressing the *P2rx7* gene or not, cell suspensions of either 1 × 10^6^ or 1 × 10^4^ 4T1-luc derived CTL (*P2rx7^+^/^+^*) cancer cells were injected to BALB/cJ mice *P2rx7^−^/^−^* (Generously provided by Niklas Rye Jørgensen, Department of Clinical Biochemistry, Rigshospitalet, Glostrup, Denmark) or to BALB/cJ *P2rx7^+^/^+^* litter mate control mice, which were housed in the same environmentally controlled conditions. *P2rx7^−^/^−^* mice were backcrossed onto the BALB/cJ background as previously described [51].

In experiments assessing the effect of *P2rx7* gene expression in mammary cancer cells on primary tumour growth and metastatic progression, 1 × 10^4^ CTL (*P2rx7*^+^/^+^), Crispr#1 or Crispr#2 (*P2rx7^−^/^−^*) 4T1-derived mammary cancer-cells (see Section 4.2) in 100 µL of a PBS solution were injected in the mammary fat pad, under isoflurane anaesthesia, of wild-type BALB/cJ mice (Janvier Labs, Saint Berthevin, France).

In experiments assessing the efficacy of P2X7 antagonism, 1 × 10^4^ CTL (*P2rx7*^+^/^+^) cells were injected in the mammary fat pad of wild-type BALB/cJ mice (Janvier Labs, Saint Berthevin, France), and treatments were randomly administrated once tumours reached 80 mm^3^. Then, 230 µL of 3 mg/mL A437079 or 100 µL of 300 nM AZ10606120 were injected intraperitoneally every two days. Primary tumours were fixed in formalin, included in paraffin, and cut in 5 µm tissue sections. Slides were deparaffinized, rehydrated and heated in citrate buffer pH 6.0 for antigenic retrieval. Immunohistochemistry was performed using a primary antibody anti-P2X7 (APR-008 Alomone Labs Ltd.) and streptavidin-biotin-peroxidase method with diaminobenzidine as the chromogen (Kit LSAB, Dakocytomation, Dako France SAS, Les Ullis, France). Slides were finally counterstained with haematoxylin. Negative controls were obtained by omission of the primary antibody or incubation with an irrelevant antibody.

### 4.16. Mathematical Model for Assessing Mammary Tumour Growth Depending on Treatments

A Gompertz model was used to describe mammary tumour growth over time. This model included three parameters, i.e., the first-order growth rate constant (k_growth_), the maximum tumour volume (V_max_) and the power coefficient γ. In addition, the effect of treatment (EFF) on tumour volume was estimated as a parameter being 0 without treatment (i.e., before treatment, in both A438079 and AZ10606120 groups, and every time in the vehicle group) and estimated when treatment was administered. Model parameters were estimated using nonlinear mixed-effects modelling. This approach allows description of the inter-subject variability in the population (mean and inter-subject standard deviations) and quantification of the association of factors of variability (referred as covariates) with inter-subject distribution of Gomperz parameters. The influence of treatment condition (A438079 or AZ10606120) was tested as a covariate on both k_growth_ and V_max_. This analysis was carried out using MonolixSuite2018 (Lixoft^®^, Antony, France). The effects of treatment and treatment conditions on each parameter were tested using the likelihood ratio test (LRT).

### 4.17. Data Presentation and Statistical Analysis

Data are displayed as median (*n* = number of cells/ independent experiments). Two-way ANOVA followed by a Dunn’s Multiple Comparison Test, Wilcoxon Signed Rank Test and Mann-Whitney rank sum test were used to compare different conditions, as indicated in the figure legends. Statistical significance is indicated as: * *p* < 0.05; ** *p* < 0.01 and *** *p* < 0.001. NS stands for not statistically different.

## 5. Conclusions

In this study, we have revealed the P2X7R in mammary cancer cells as a key molecular determinant of cancer cell invasiveness and mammary tumour growth, and as a possible target for pharmacological interventions.

## Figures and Tables

**Table 1:** Sequences of conventional Polymerase Chain Reactions (PCR) and quantitative Polymerase Chain Reactions (qPCR) primers and expected amplicon size.

Mouse Genes	Applications	Corresponding Proteins	Forward Primers (5′→3′)	Reverse Primers (5′→3′)	Amplicon—Expected Size (bp)
Primers for conventional PCR					
*mP2rx1*	PCR	P2X1	CCTCAAGTGGCCTTATCAGC	GGTACCATTCACCTCCTCCA	467
*mP2rx2*	PCR	P2X2	ACGTTCATGAACAAAAACAAG	TCAAAGTTGGGCCAAACCTTTGG	360
*mP2rx3*	PCR	P2X3	GGACGCTATGCCAACAGAGT	ACGTCCCCTACCCTCAAGAT	461
*mP2rx4*	PCR	P2X4	GCGTCTGTGAAGACCTGTGA	GCCTTTCCAAACACGATGAT	521
*mP2rx5*	PCR	P2X5	TCCCGGATGGCGAGTGTTCAG	GATGGGGCAGTAGAGATTGGTGGAG	323
*mP2rx6*	PCR	P2X6	AACTGGGAACATGGCTTCTG	CACCAGCTCCAGATCTCACA	516
*mP2rx7*	PCR	P2X7	TGCACATGATCGTCTTTTCC	GGCAAGATGTTTCTCGTGGT	532
Primers for qPCR					
*mP2rx7*	qPCR	P2X7	AGCACGAATTATGGCACCGT	CCCCACCCTCTGTGACATTCT	172
*mCDH1*	qPCR	E-Cadherin	CAGTTCCGAGGTCTACACCTT	TGAATCGGGAGTCTTCCGAAAA	131
*mGusb*	qPCR	β-Glucuronidase	TATGGAGCAGACGCAATCCC	TTCGTCATGAAGTCGGCGAA	164
*mSnai1*	qPCR	Snail1	CACACGCTGCCTTGTGTCT	GGTCAGCAAAAGCACGGTT	133
*mTwist*	qPCR	Twist	TTCTCGGTCTGGAGGATGGA	TCTCTGGAAACAATGACATCTAGG	110
*mZeb1*	qPCR	Zeb1	ACCGCCGTCATTTATCCTGAG	CATCTGGTGTTCCGTTTTCATCA	91
*mZO1*	qPCR	ZO1	GCCTCAGAAATCCAGCTTCTCGAA	GCAGCTAGCCAGTGTACAGTATAC	194

## Supplementary Materials

The following are available online at https://www.mdpi.com/2072-6694/12/9/2342/s1: Figure S1: P2X7R receptor is functional in 4T1 mammary cancer cells; Figure S2: P2X7R receptor promotes 3D cancer cell invasiveness of MDA-MB435s human cancer cells; Figure S3: P2X7R receptor promotes invadopodial activity of extracellular matrix degradation in MDA-MB-435s human cancer cells; Figure S4: Antagonism of P2X7R reduces primary tumour growth; Figure S5: Full and uncropped blots shown in Figure 2b,f; Figure S6: Full and uncropped blots shown in Figure S3a; Figure S7: Full and uncropped blots shown in Figure 3d,f; Video S1: Control condition in absence of agonist or antagonist (vehicle); Video S2: stimulation with 300 µM BzATP; Video S3: incubation with 10 µM A438079; Video S4: incubation with 300 µM BzATP in the presence of 10 µM A438079.

## References

[1] Jiang L.H., Baldwin J.M., Roger S., Baldwin S.A. (2013). Insights into the Molecular Mechanisms Underlying Mammalian P2X7 Receptor Functions and Contributions in Diseases, Revealed by Structural Modeling and Single Nucleotide Polymorphisms. Front. Pharmacol..

[2] Roger S., Gillet L., Baroja-Mazo A., Surprenant A., Pelegrin P. (2010). C-terminal calmodulin-binding motif differentially controls human and rat P2X7 receptor current facilitation. J. Biol. Chem..

[3] Di Virgilio F., Dal Ben D., Sarti A.C., Giuliani A.L., Falzoni S. (2017). The P2X7 Receptor in Infection and Inflammation. Immunity.

[4] Roger S., Jelassi B., Couillin I., Pelegrin P., Besson P., Jiang L.H. (2015). Understanding the roles of the P2X7 receptor in solid tumour progression and therapeutic perspectives. Biochim. Biophys. Acta.

[5] Li X., Qi X., Zhou L., Catera D., Rote N.S., Potashkin J., Abdul-Karim F.W., Gorodeski G.I. (2007). Decreased expression of P2X7 in endometrial epithelial pre-cancerous and cancer cells. Gynecol. Oncol..

[6] Li X., Qi X., Zhou L., Fu W., Abdul-Karim F.W., Maclennan G., Gorodeski G.I. (2009). P2X(7) receptor expression is decreased in epithelial cancer cells of ectodermal, uro-genital sinus, and distal paramesonephric duct origin. Purinergic Signal..

[7] Adinolfi E., Callegari M.G., Cirillo M., Pinton P., Giorgi C., Cavagna D., Rizzuto R., Di Virgilio F. (2009). Expression of the P2X7 receptor increases the Ca^2+^ content of the endoplasmic reticulum, activates NFATc1, and protects from apoptosis. J. Biol. Chem..

[8] Adinolfi E., Cirillo M., Woltersdorf R., Falzoni S., Chiozzi P., Pellegatti P., Callegari M.G., Sandona D., Markwardt F., Schmalzing G. (2010). Trophic activity of a naturally occurring truncated isoform of the P2X7 receptor. FASEB J..

[9] Adinolfi E., Raffaghello L., Giuliani A.L., Cavazzini L., Capece M., Chiozzi P., Bianchi G., Kroemer G., Pistoia V., Di Virgilio F. (2012). Expression of P2X7 Receptor Increases In Vivo Tumor Growth. Cancer Res..

[10] Takai E., Tsukimoto M., Harada H., Kojima S. (2014). Autocrine signaling via release of ATP and activation of P2X7 receptor influences motile activity of human lung cancer cells. Purinergic Signal..

[11] Jelassi B., Anchelin M., Chamouton J., Cayuela M.L., Clarysse L., Li J., Gore J., Jiang L.H., Roger S. (2013). Anthraquinone emodin inhibits human cancer cell invasiveness by antagonizing P2X7 receptors. Carcinogenesis.

[12] Jelassi B., Chantome A., Alcaraz-Perez F., Baroja-Mazo A., Cayuela M.L., Pelegrin P., Surprenant A., Roger S. (2011). P2X(7) receptor activation enhances SK3 channels- and cystein cathepsin-dependent cancer cells invasiveness. Oncogene.

[13] Giannuzzo A., Pedersen S.F., Novak I. (2015). The P2X7 receptor regulates cell survival, migration and invasion of pancreatic ductal adenocarcinoma cells. Mol. Cancer.

[14] Giannuzzo A., Saccomano M., Napp J., Ellegaard M., Alves F., Novak I. (2016). Targeting of the P2X7 receptor in pancreatic cancer and stellate cells. Int. J. Cancer.

[15] Qiu Y., Li W.H., Zhang H.Q., Liu Y., Tian X.X., Fang W.G. (2014). P2X7 mediates ATP-driven invasiveness in prostate cancer cells. PLoS ONE.

[16] Roger S., Pelegrin P. (2011). P2X7 receptor antagonism in the treatment of cancers. Expert Opin. Investig. Drugs.

[17] Coutinho-Silva R., Stahl L., Cheung K.K., de Campos N.E., de Oliveira Souza C., Ojcius D.M., Burnstock G. (2005). P2X and P2Y purinergic receptors on human intestinal epithelial carcinoma cells: Effects of extracellular nucleotides on apoptosis and cell proliferation. Am. J. Physiol. Gastrointest. Liver Physiol..

[18] Aymeric L., Apetoh L., Ghiringhelli F., Tesniere A., Martins I., Kroemer G., Smyth M.J., Zitvogel L. (2010). Tumor cell death and ATP release prime dendritic cells and efficient anticancer immunity. Cancer Res..

[19] Ghiringhelli F., Apetoh L., Tesniere A., Aymeric L., Ma Y., Ortiz C., Vermaelen K., Panaretakis T., Mignot G., Ullrich E. (2009). Activation of the NLRP3 inflammasome in dendritic cells induces IL-1beta-dependent adaptive immunity against tumors. Nat. Med..

[20] Slater M., Danieletto S., Barden J.A. (2005). Expression of the apoptotic calcium channel P2X7 in the glandular epithelium. J. Mol. Histol..

[21] Park M., Kim J., Phuong N.T.T., Park J.G., Park J.H., Kim Y.C., Baek M.C., Lim S.C., Kang K.W. (2019). Involvement of the P2X7 receptor in the migration and metastasis of tamoxifen-resistant breast cancer: Effects on small extracellular vesicles production. Sci. Rep..

[22] Xia J., Yu X., Tang L., Li G., He T. (2015). P2X7 receptor stimulates breast cancer cell invasion and migration via the AKT pathway. Oncol. Rep..

[23] Brisson L., Reshkin S.J., Gore J., Roger S. (2012). pH regulators in invadosomal functioning: Proton delivery for matrix tasting. Eur. J. Cell Biol..

[24] Aslakson C.J., Miller F.R. (1992). Selective events in the metastatic process defined by analysis of the sequential dissemination of subpopulations of a mouse mammary tumor. Cancer Res..

[25] Khalid M., Brisson L., Tariq M., Hao Y., Guibon R., Fromont G., Mortadza S.A.S., Mousawi F., Manzoor S., Roger S. (2017). Carcinoma-specific expression of P2Y11 receptor and its contribution in ATP-induced purinergic signalling and cell migration in human hepatocellular carcinoma cells. Oncotarget.

[26] Brisson L., Driffort V., Benoist L., Poet M., Counillon L., Antelmi E., Rubino R., Besson P., Labbal F., Chevalier S. (2013). NaV1.5 Na^+^ channels allosterically regulate the NHE-1 exchanger and promote the activity of breast cancer cell invadopodia. J. Cell Sci..

[27] Linder S., Wiesner C., Himmel M. (2011). Degrading devices: Invadosomes in proteolytic cell invasion. Annu. Rev. Cell Dev. Biol..

[28] Maldonado M.D.M., Dharmawardhane S. (2018). Targeting Rac and Cdc42 GTPases in Cancer. Cancer Res..

[29] Clark A.G., Vignjevic D.M. (2015). Modes of cancer cell invasion and the role of the microenvironment. Curr. Opin. Cell Biol..

[30] Mierke C.T. (2019). The matrix environmental and cell mechanical properties regulate cell migration and contribute to the invasive phenotype of cancer cells. Rep. Prog. Phys..

[31] Di Virgilio F., Sarti A.C., Falzoni S., De Marchi E., Adinolfi E. (2018). Extracellular ATP and P2 purinergic signalling in the tumour microenvironment. Nat. Rev. Cancer.

[32] Pellegatti P., Raffaghello L., Bianchi G., Piccardi F., Pistoia V., Di Virgilio F. (2008). Increased level of extracellular ATP at tumor sites: In vivo imaging with plasma membrane luciferase. PLoS ONE.

[33] Raffaghello L., Chiozzi P., Falzoni S., Di Virgilio F., Pistoia V. (2006). The P2X7 receptor sustains the growth of human neuroblastoma cells through a substance P-dependent mechanism. Cancer Res..

[34] Di Virgilio F., Chiozzi P., Falzoni S., Ferrari D., Sanz J.M., Venketaraman V., Baricordi O.R. (1998). Cytolytic P2X purinoceptors. Cell Death Differ..

[35] Slater M., Danieletto S., Gidley-Baird A., Teh L.C., Barden J.A. (2004). Early prostate cancer detected using expression of non-functional cytolytic P2X7 receptors. Histopathology.

[36] Gorodeski G.I. (2009). P2X7-mediated chemoprevention of epithelial cancers. Expert Opin. Ther. Targets.

[37] Adinolfi E., Callegari M.G., Ferrari D., Bolognesi C., Minelli M., Wieckowski M.R., Pinton P., Rizzuto R., Di Virgilio F. (2005). Basal activation of the P2X7 ATP receptor elevates mitochondrial calcium and potential, increases cellular ATP levels, and promotes serum-independent growth. Mol. Biol. Cell.

[38] Bours M.J., Swennen E.L., Di Virgilio F., Cronstein B.N., Dagnelie P.C. (2006). Adenosine 5′-triphosphate and adenosine as endogenous signaling molecules in immunity and inflammation. Pharmacol. Ther..

[39] Adinolfi E., Capece M., Franceschini A., Falzoni S., Giuliani A.L., Rotondo A., Sarti A.C., Bonora M., Syberg S., Corigliano D. (2015). Accelerated tumor progression in mice lacking the ATP receptor P2X7. Cancer Res..

[40] Hofman P., Cherfils-Vicini J., Bazin M., Ilie M., Juhel T., Hebuterne X., Gilson E., Schmid-Alliana A., Boyer O., Adriouch S. (2015). Genetic and pharmacological inactivation of the purinergic P2RX7 receptor dampens inflammation but increases tumor incidence in a mouse model of colitis-associated cancer. Cancer Res..

[41] Holzel M., Tuting T. (2016). Inflammation-Induced Plasticity in Melanoma Therapy and Metastasis. Trends Immunol..

[42] Janakiram N.B., Rao C.V. (2014). The role of inflammation in colon cancer. Adv. Exp. Med. Biol..

[43] Parkin D.M., Bray F., Ferlay J., Pisani P. (2005). Global cancer statistics, 2002. CA Cancer J. Clin..

[44] Voduc K.D., Cheang M.C., Tyldesley S., Gelmon K., Nielsen T.O., Kennecke H. (2010). Breast cancer subtypes and the risk of local and regional relapse. J. Clin. Oncol..

[45] Zhang Y., Cheng H., Li W., Wu H., Yang Y. (2019). Highly-expressed P2X7 receptor promotes growth and metastasis of human HOS/MNNG osteosarcoma cells via PI3K/Akt/GSK3beta/beta-catenin and mTOR/HIF1alpha/VEGF signaling. Int. J. Cancer.

[46] Lou Y., Preobrazhenska O., auf dem Keller U., Sutcliffe M., Barclay L., McDonald P.C., Roskelley C., Overall C.M., Dedhar S. (2008). Epithelial-mesenchymal transition (EMT) is not sufficient for spontaneous murine breast cancer metastasis. Dev. Dyn..

[47] Saitoh M. (2018). Involvement of partial EMT in cancer progression. J. Biochem..

[48] Guo Q., Betts C., Pennock N., Mitchell E., Schedin P. (2017). Mammary Gland Involution Provides a Unique Model to Study the TGF-beta Cancer Paradox. J. Clin. Med..

[49] Driffort V., Gillet L., Bon E., Marionneau-Lambot S., Oullier T., Joulin V., Collin C., Pages J.C., Jourdan M.L., Chevalier S. (2014). Ranolazine inhibits NaV1.5-mediated breast cancer cell invasiveness and lung colonization. Mol. Cancer.

[50] Bon E., Driffort V., Gradek F., Martinez-Caceres C., Anchelin M., Pelegrin P., Cayuela M.L., Marionneau-Lambot S., Oullier T., Guibon R. (2016). SCN4B acts as a metastasis-suppressor gene preventing hyperactivation of cell migration in breast cancer. Nat. Commun..

[51] Syberg S., Petersen S., Beck Jensen J.E., Gartland A., Teilmann J., Chessell I., Steinberg T.H., Schwarz P., Jorgensen N.R. (2012). Genetic Background Strongly Influences the Bone Phenotype of P2X7 Receptor Knockout Mice. J. Osteoporos..

