# Supplementary material for: P2X7 Receptor Promotes Mouse Mammary Cancer Cell Invasiveness and Tumour Progression, and Is a Target for Anticancer Treatment
Source: Cancers (Basel). 2020 Aug 19;12(9):2342. doi: 10.3390/cancers12092342 (PMC7565976; doi:10.3390/cancers12092342)
Supplement: Supplementary file 1 [file cancers-12-02342-s001.zip › cancers-877261 Supplementary Material.pdf]

# Supplementary Material: P2X7 Receptor Promotes Mouse Mammary Cancer Cell Invasiveness and Tumour Progression, and Is a Target for Anticancer Treatment

**Lucie Brisson, Stéphanie Chadet, Osbaldo Lopez-Charcas, Bilel Jelassi, David Ternant, Julie Chamouton, Stéphanie Lerondel, Alain Le Pape, Isabelle Couillin, Aurélie Gombault, Fabrice Trovero, Stéphan Chevalier, Pierre Besson, Lin-Hua Jiang and Sébastien Roger**

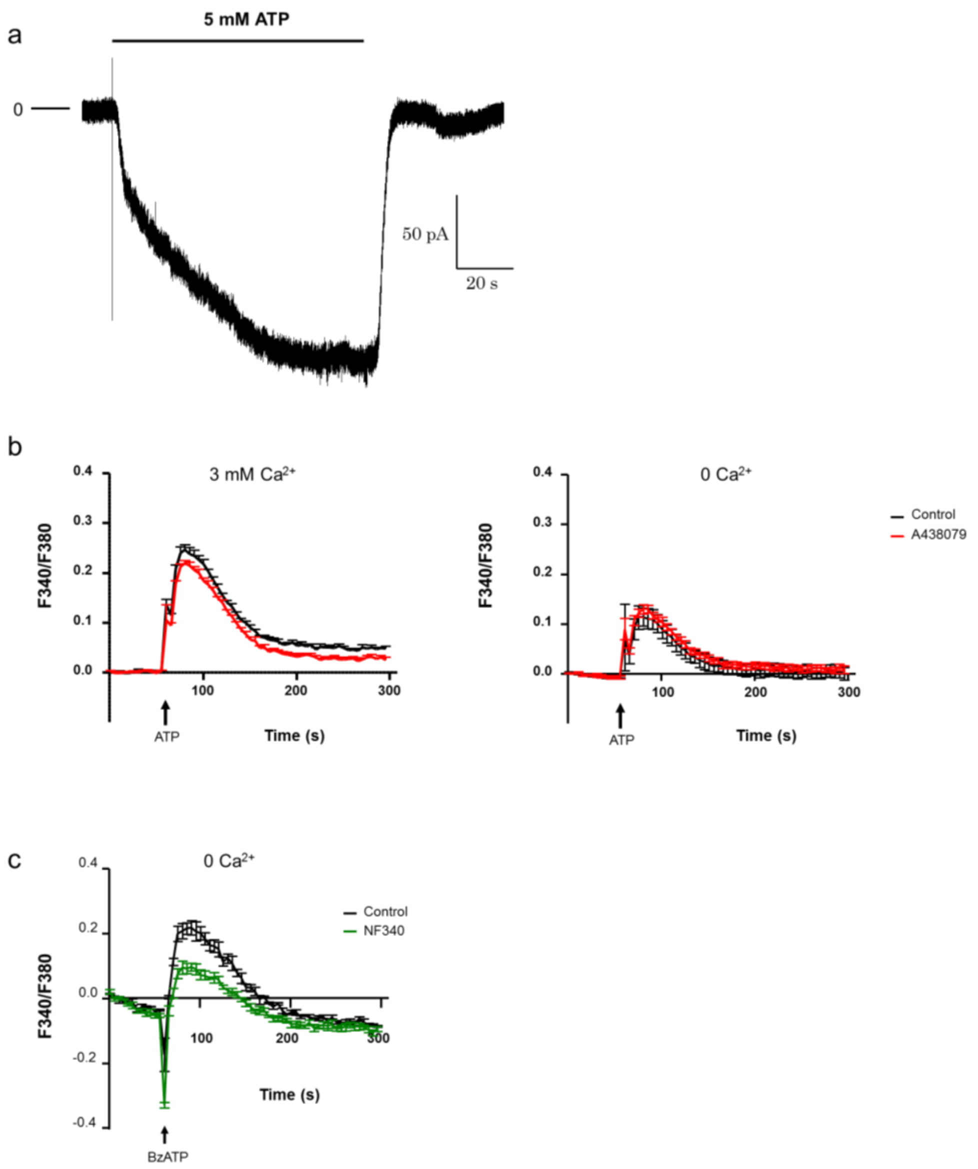


**Figure S1.** P2X7 receptor is functional in 4T1 mammary cancer cells. (**a**) Representative whole-cell patch clamp recordings from 4T1 mouse mammary cancer cells which were held at a membrane potential of −60 mV. A 60 s-long application of 5 mM ATP produced a facilitating inward current. "0" indicates the zero level current. (**b**) Intracellular free $Ca^{2+}$ levels were monitored in 4T1 mouse mammary cancer cells by the use of Fura2 fluorescence, expressed as a ratio of the 510 nm emission when excited at 340 nm and 380 nm ($F_{340/F380}$), thus indicating the level of free $Ca^{2+}$ as a function of time, $Ca^{2+}$ response to 3 mM ATP stimulation, in the absence (black traces, control) or presence (red traces) of P2X7 antagonist A438079 (10 μM). This same protocol was performed in the presence of 3

mM extracellular $Ca^{2+}$ (left traces) or in the absence of extracellular $Ca^{2+}$ (right traces). These data are averaged from 3 independent experiments (**c**). Similar protocol as shown in (**a**), in the absence of extracellular $Ca^{2+}$ (right traces), after stimulation with 300 μM BzATP at the time indicated by the arrow, in the absence (black traces, control, vehicle) or presence (green traces) of P2Y11 antagonist NF340 (10 μM). These data are representative from 4 independent experiments.

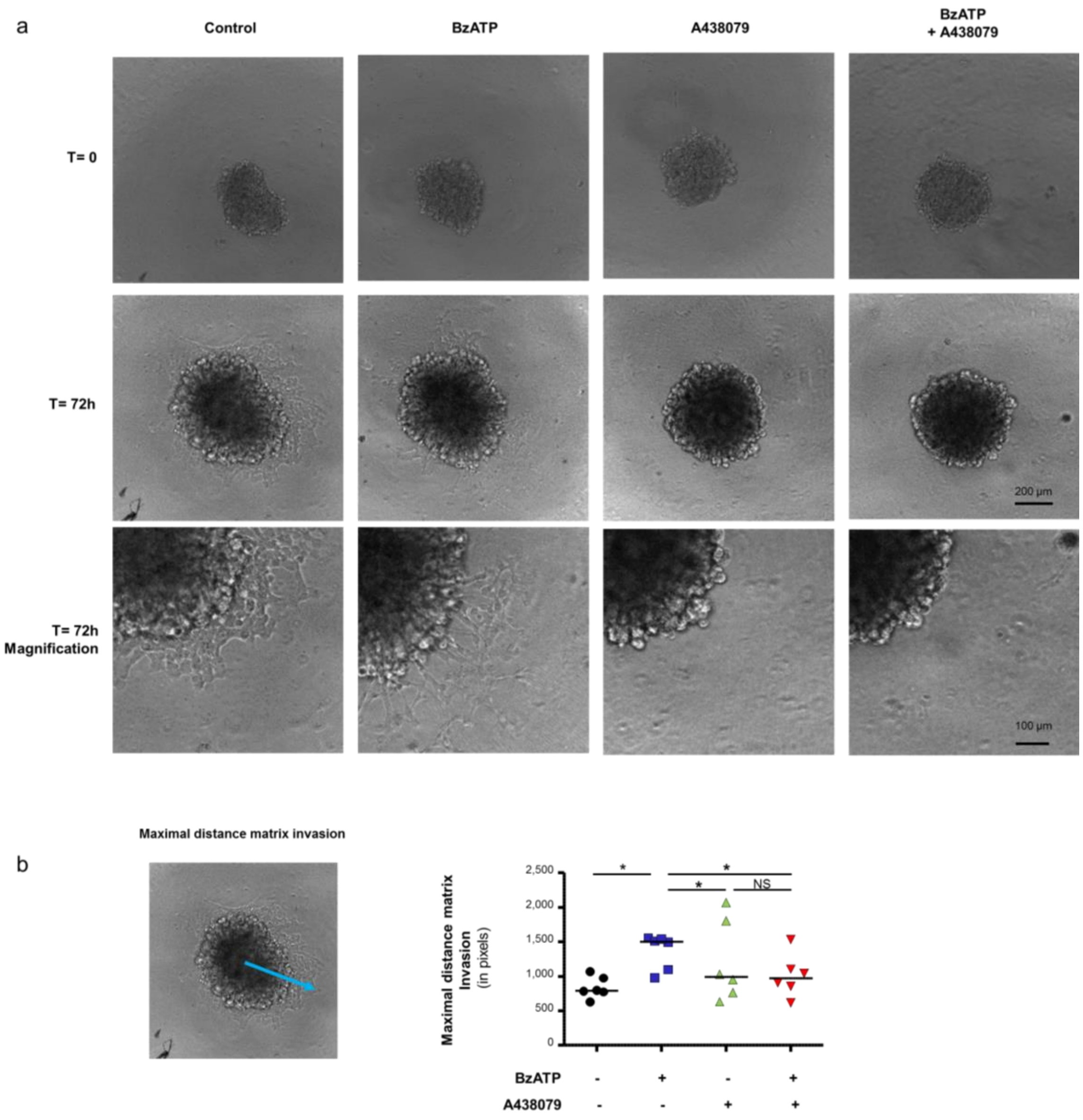


**Figure 2.** P2X7 receptor promotes 3D-cancer cell invasiveness of MDA-MB-435s human cancer cells. (**a**) Representative images showing the morphology and size of cancer cell spheroids assessed before (T = 0 h) or after 72 h treatment (T = 72 h) in control condition (vehicle) or with BzATP (300 μM) in the absence or presence of A438079 (10 μM). P2X7 activity promotes cancer cell escape from spheroids and invasion of extracellular matrix. Scale bar, 200 μm. Magnification, scale bar 100 μm. (**b**) Assessment of the maximal distance invaded by cancer cells from the center of spheroids (expressed in pixels). Data are from 6 independent experiments. * indicates a statistically significant difference at $p < 0.05$ using Dunn's Multiple Comparison test. NS stands for no statistical significant difference.

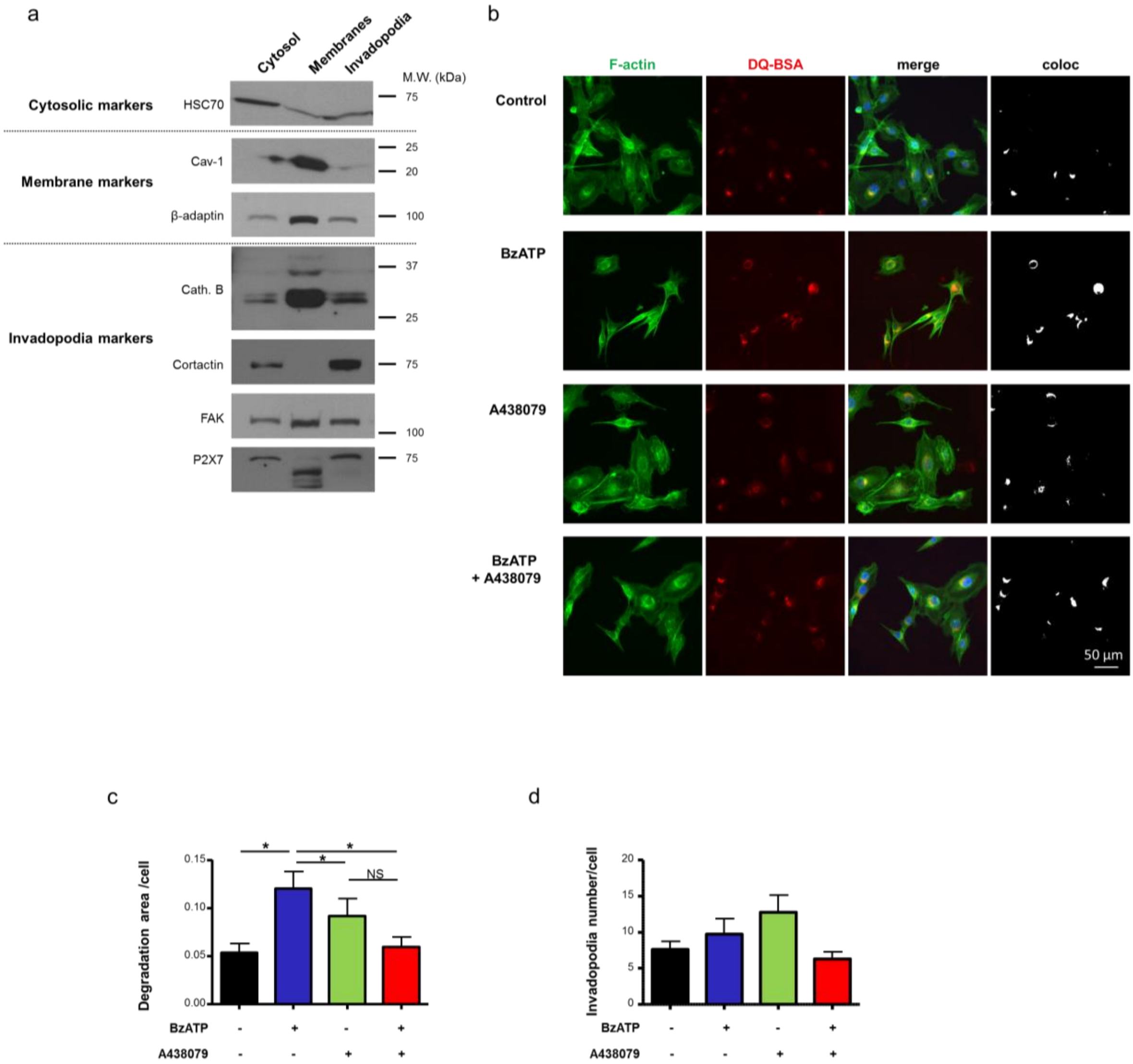


**Figure S3.** P2X7 receptor promotes invadopodial activity of extracellular matrix degradation in MDA-MB-435s human cancer cells. (**a**) Invadopodia of MDA-MB-435s cells, entrapped into a 2%-gelatin matrix, were fractionated and separated from cytosol and membranes-enriched fractions. The quality of the fractions was assessed by western blotting, using cytosolic (HSC70), membrane (caveolin-1, β-adaptin) and invadopodia (cathepsin B, cortactin, Focal Adhesion Kinase) markers. P2X7 proteins were found to be present in the membrane fractions and enriched in the invadopodia fraction. These results are representative of 5 independent experiments. The full blots images can be found in Figure S6. (**b**) The invadopodial activity was assessed as being F-actin foci (green labelling, phalloidin-488) co-localised with focused proteolytic activities (red labelling, DQ-BSA proteolysis) from MDA-MB435s cells grown for 24h on Matrigel® containing DQ-BSA in control condition (vehicle), or stimulated with 300 µM BzATP, in the presence or absence of 10 µM A438079. "Coloc" indicates co-localization of degradative activity and F-actin foci, which appears as white pixels. Scale bar, 50 µm. (**c**) The number of degradation areas per cell was counted in 20 images, from 5 independent experiments. * indicates a statistically significant difference at $p < 0.05$ using Dunn's Multiple Comparison test. NS stands for no statistical significant difference. (**d**) The number of invadopodia (identified as being F-actin foci co-localized with matrix degradation spots) was counted per cell in the same experiments as shown in (**c**). There was no statistically significant difference among the four treatment conditions.

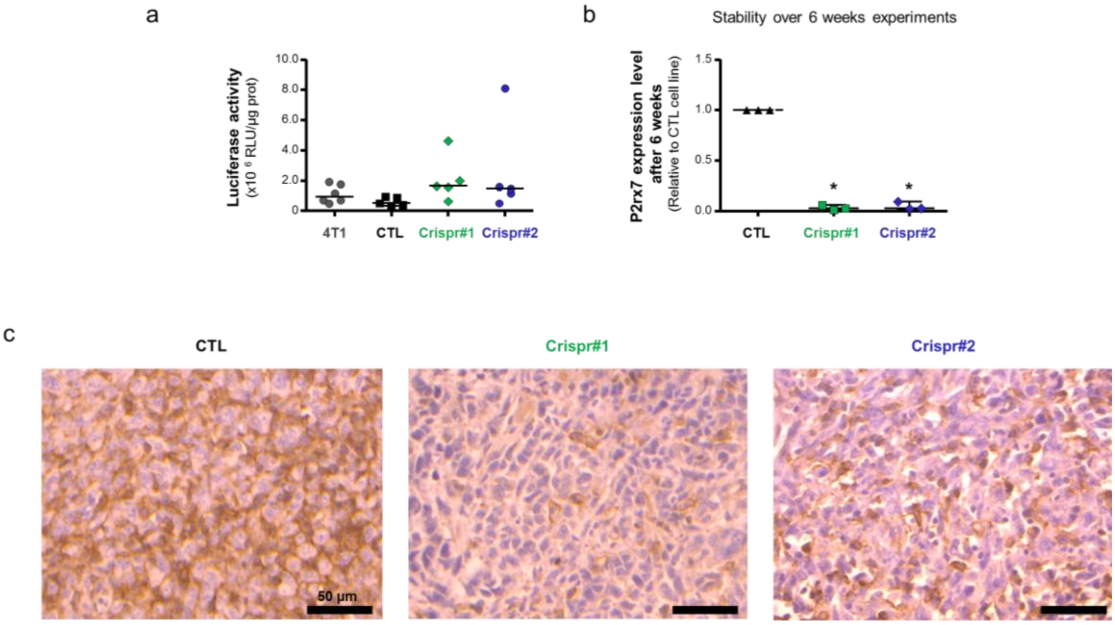


**Figure S4.** Antagonism of P2X7 reduces primary tumour growth. (**a**) Comparison of luciferase activity between the parental 4T1 cell line and the three clonal cell lines generated as indicated in Section 4 using the CRISPR technique, CTL, Crispr#1 and Crispr#2 cell lines. All cell lines demonstrated similar luciferase activity, comparable to the parental 4T1 cell line. (**b**) Stability over 6 weeks of the Crispr#1 and Crispr#2 clonal cell lines for the knock-down of the P2rx7 gene was assessed by RT-qPCR, for the same duration as the in vivo experiment. * indicates a statistical difference from CTL cell line at $p < 0.05$ using Dunn's Multiple Comparison test. ($n$ = 3 independent experiments). (**c**) P2X7 immunodetection (brown staining) in primary mammary tumours coming from the implantation of CTL, Crispr#1 or Crispr#2 cell lines and showing a significant reduction in the P2X7 staining in the two Crispr cell lines.

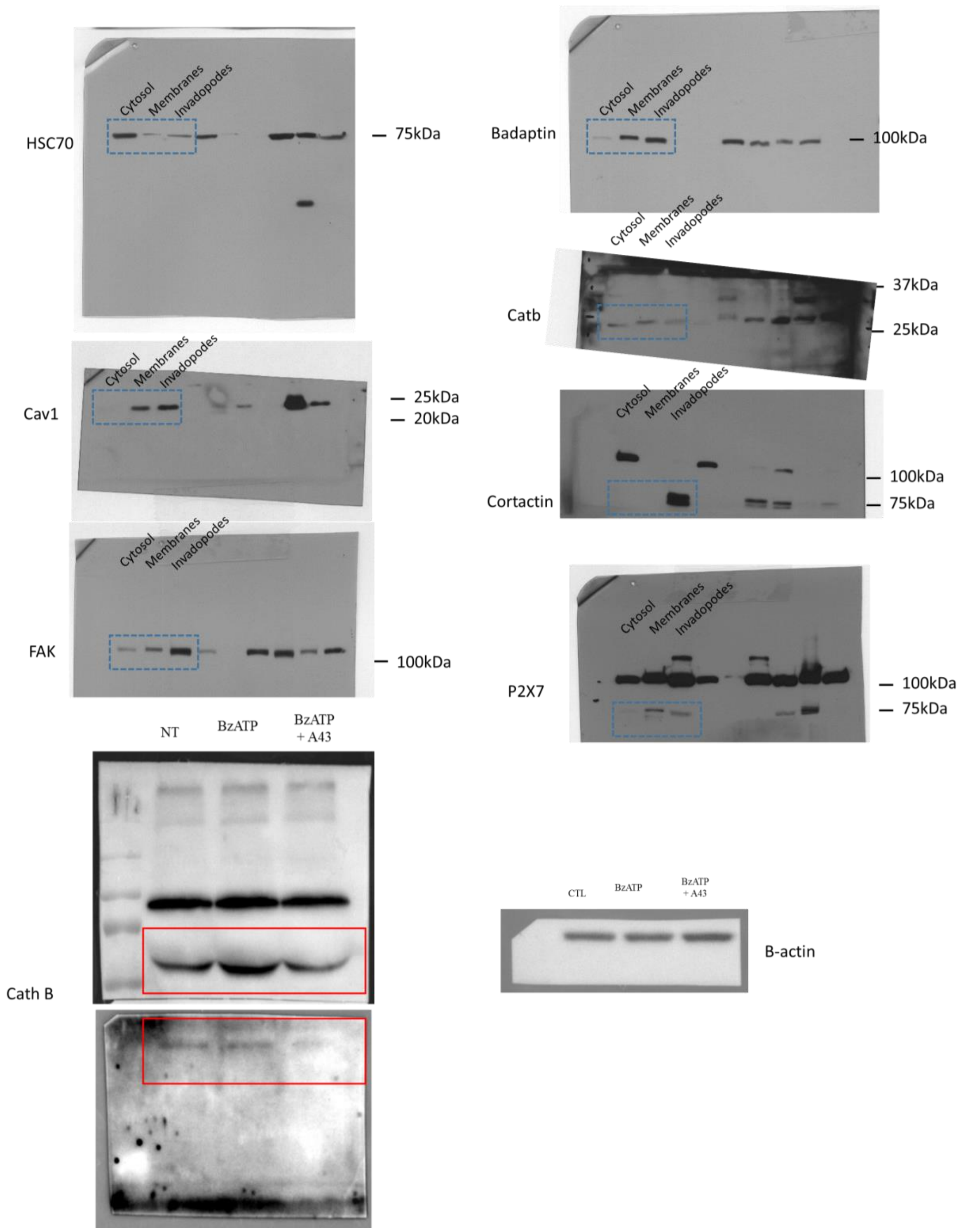


**Figure S5:** Full and uncropped blots shown in Figure 2b,f.

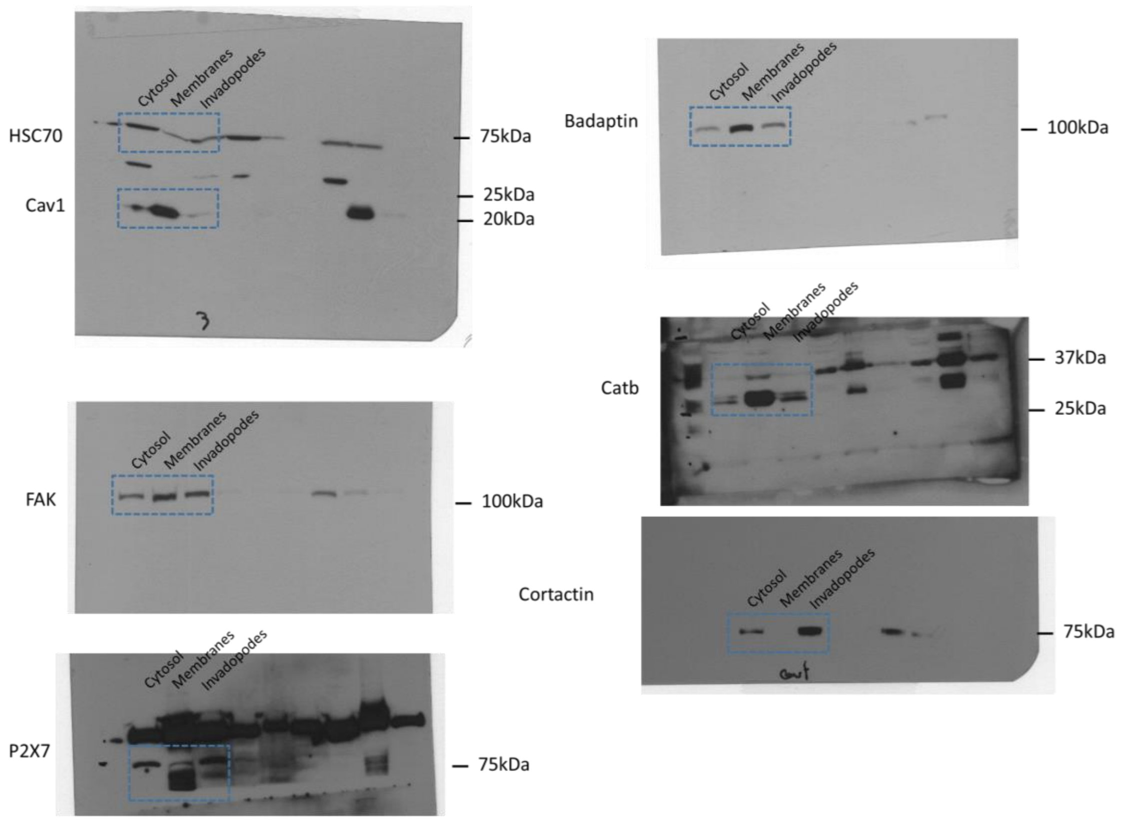


**Figure S6.** Full and uncropped blots shown in Figure S3a.

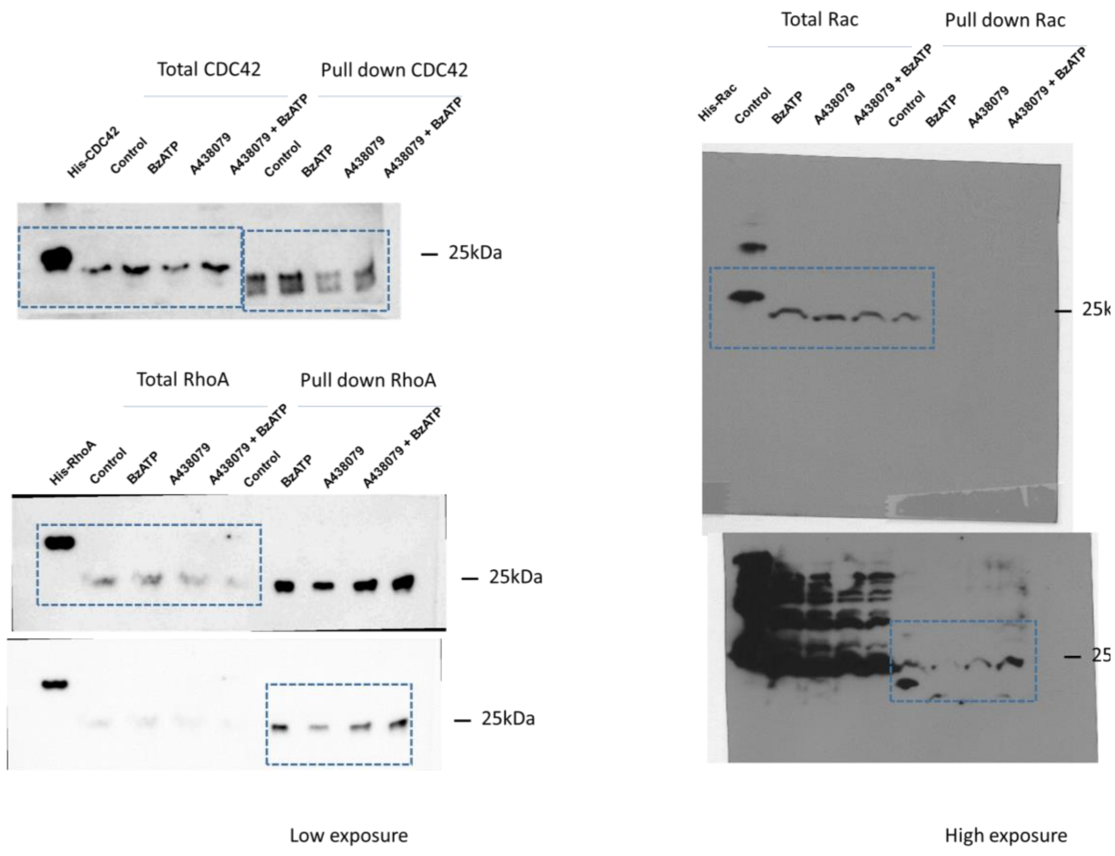

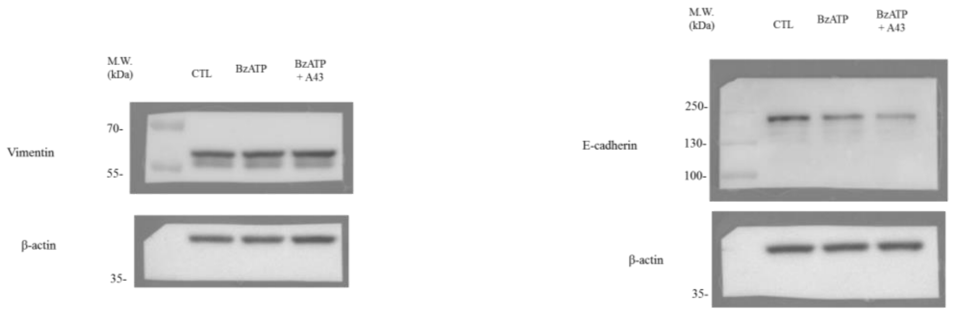


**Figure S7.** Full and uncropped blots shown in Figure 3d,f.

Videos S1–S4 (separate .zip file "Videos S1–S4"): Time-lapse microscopy movies (7 frames/s) assessing filopodia remodelling in 4T1 cells transfected with LifeAct plasmid.

**Video S1**: Control condition in absence of agonist or antagonist (vehicle).

**Video S2**: stimulation with 300 μM BzATP.

**Video S3**: incubation with 10 μM A438079.

**Video S4**: incubation with 300 μM BzATP in presence of 10 μM A438079.
